# Pharmacokinetic and Metabolism Studies of 12-Riboside-Pseudoginsengenin DQ by UPLC-MS/MS and UPLC-QTOF-MS^E^

**DOI:** 10.3390/molecules23102499

**Published:** 2018-09-29

**Authors:** Zhenzhou Wang, Hongqiang Lin, Hailin Zhu, Na Yang, Baisong Zhou, Cuizhu Wang, Pingya Li, Jinping Liu

**Affiliations:** School of Pharmaceutical Sciences, Jilin University, Fujin Road 1266, Changchun 130021, China; wzz16@mails.jlu.edu.cn (Z.W.); linhq17@mails.jlu.edu.cn (H.L.); 13578965875@163.com (H.Z.);yangn0227@163.com (N.Y.); zhoubs16@mails.jlu.edu.cn (B.Z.); wangcz15@mails.jlu.edu.cn (C.W.)

**Keywords:** 12-riboside-pseudoginsengenin DQ, pharmacokinetics, metabolism, UPLC-MS

## Abstract

Pharmacokinetic and metabolism studies of 12-riboside-pseudoginsengenin DQ (RPDQ), a novel ginsenoside with an anti-cancer effect, were carried out, aiming at discussing the characteristics of the ginsenoside with glycosylation site at C-12. In the pharmacokinetic analysis, we developed and validated a method by UPLC-MS to quantify RPDQ in rat plasma. In the range of 5–1000 ng/mL, the assay was linear (R^2^ > 0.9966), with the LLOQ (lower limit of quantification) being 5 ng/mL. The LOD (limit of detection) was 1.5 ng/mL. The deviations of intra-day and inter-day, expressed as relative standard deviation (RSD), were ≤ 3.51% and ≤ 5.41% respectively. The accuracy, expressed as relative error (RE), was in the range –8.82~3.47% and –5.61~2.87%, respectively. The recoveries were in the range 85.66~92.90%. The method was then applied to a pharmacokinetic study in rats intragastrically administrated with 6, 12, and 24 mg/kg RPDQ. The results showed that RPDQ exhibited slow oral absorption (*T*_max_ = 7.0 h, 7.5 h, and 7.0 h, respectively), low elimination (*t*_1/2_ = 12.59 h, 12.83 h, and 13.74 h, respectively) and poor absolute bioavailability (5.55, 5.15, and 6.08%, respectively). Moreover, the investigation of metabolites were carried out by UPLC-QTOF-MS. Thirteen metabolites of RPDQ were characterized from plasma, bile, urine, and feces of rats. Some metabolic pathways, including oxidation, acetylation, hydration, reduction, hydroxylation, glycine conjugation, sulfation, phosphorylation, glucuronidation, glutathione conjugation, and deglycosylation, were profiled. In general, both the rapid quantitative method and a good understanding of the characteristics of RPDQ in vivo were provided in this study.

## 1. Introduction

Ginseng, a traditional Chinese medicine, has been widely used. Ginsenosides are the main active ingredient [1,2]. According to the skeleton of sapogenin, ginsenosides can be divided into tetracyclic and pentacyclic triterpene types [3,4]. The former type could be divided into dammarane-subtype and ocotillol-subtype saponins [5]. It is worth mentioning that ocotillol-type saponin (20, 24-epoxyside) has many high activities such as neuroprotective, antiinflammatory, antibacterial, and antitumor effect [6,7,8,9]. The discovery of this type showed that the natural ones were mainly existed in American ginseng, Vietnam ginseng, or Panax japonicas [10,11,12]. Recently, the semisynthesis has been receiving more and more attention aiming to increase the yields or obtain the novel ocotillol-type saponin [13,14,15,16]. In our study, a novel ocotillol-type ginsenoside, named 12-riboside-pseudoginsengenin DQ (RPDQ), was semi-synthesized successfully by glycosylation reaction of pseudoginsengenin DQ. It was structurally characterized as 20*S*, 24*S*-12-*O*-*β*-d-ribofuranosyl-dammar-20, 24-epoxy-3*β*, 12*β*, 25-triol with HR-MS and NMR analysis (Supplementary Material 1) and showed a significant anticancer activity against S180 cells, A549 cells, and SPC-A-1 cells in vitro (Supplementary Material 2).

So far, there have been some pharmacokinetic study on sapogenins and ginsenosides. Among the most commonly measured pharmacokinetic parameters, some of the characteristics such as elimination half-life (*t*_1/2_) and bioavailability (*F*) are various for different saponins after intragastric administration. For example, protopanaxdiol takes less time (*t*_1/2_: 1.48 h) to reach half the concentration of the original value and a higher bioavailability (F: 36.8%) [17]. However, pseudoginsengenin DQ, the ocotillol-type sapogenin prepared by oxidative cyclization of protopanaxadiol, has a longer biological half time (*t*_1/2_: 5.97 h) [18]. On the other hand, for different structure types of ginsenoside, there are many differences in pharmacokinetic characteristics. Ginsenosides Rb_1_, Rb_2_, and Rb_3_, with the glycoside position being C-3, had a longer biological half time (*t*_1/2_: 9.8 h, 23.1 h, and 21.1 h, respectively) and extremely low bioavailability (0.78, 0.08, and 0.52%, respectively) [19]. Ginsenoside CK, with the glycoside position being C-20, has a better bioavailability (4.30%) [20]. In addition, ginsenosides Rg_1_ and Rh_1_, with the glycoside position being C-6, also have poor bioavailabilities (1.33 and 1.01%) [21,22]. Moreover, the *t*_1/2_ (0.43 h) of Rh_1_ was substantially low [23]. Consequently, the pharmacokinetic characteristics are closely related to the structures of compounds. The glycoside positions of ginsenosides found in pharmacokinetic studies have all been C-3, C-6, and C-20, while those found in pharmacokinetic studies on ginsenosides with glycosylation site at C-12 remain blank.

In this study, RPDQ was used to carry out pharmacokinetic and metabolism studies. A sensitive and rapid UPLC-MS/MS quantification method has been developed and validated. The method was then applied to a pharmacokinetic study in rats administrated with RPDQ. Metabolic characterization of RPDQ was also carried out to explicate the dynamic process of RPDQ in vivo. The aim was to discuss the pharmacokinetic and metabolism characteristics of the ginsenoside with a glycosylation site at C-12. In general, a rapid quantitative method and a good understanding of the characteristics of RPDQ in vivo are provided in this study.

## 2. Results

### 2.1. Pharmacokinetic Study

#### 2.1.1. Method Development

**Figure 1:**
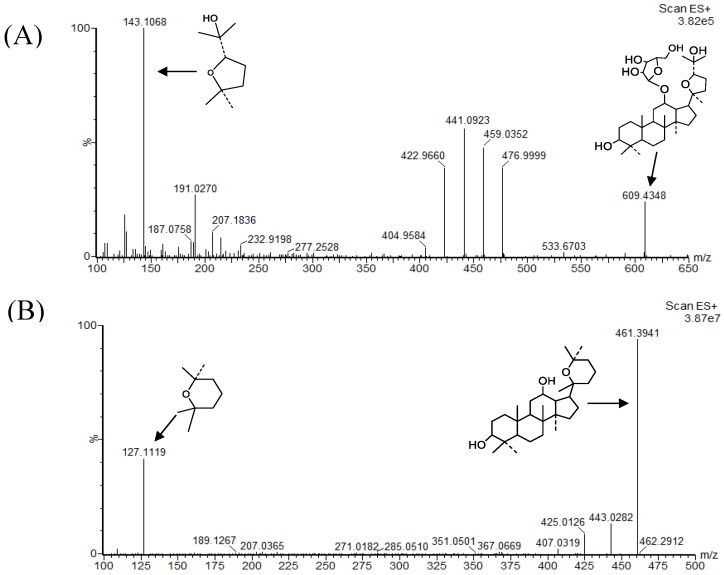
Mass spectra and the proposed patterns of fragmentation of RPDQ (**A**) and internal standard (IS) (**B**).

Different sample pre-treatment methods including protein precipitation, liquid–liquid, or solid-phase extraction were comparatively investigated to minimize the matrix effect and increase the extraction recovery. As a result, the method of precipitating protein by methanol was chosen. In order to increase the sensitivity of the RPDQ and IS, 0.1% formic acid was added to the mobile phase. In the current conditions, there was no significant signal enhancement or inhibition was found. Several possible internal standards, including 20*R*-panaxadiol, ocotillol and protopanaxdiol, were tested. Ocotillol was considered as IS, while the retention time of it was not satisfactory under the analysis condition. Protopanaxdiol had a low sensitivity. The extraction efficiency and chromatographic behavior of 20*R*-panaxadiol was similar to RPDQ, so it was selected as the IS. Due to the similar structures, RPDQ, and 20*R*-panaxadiol could all produce strong signals in the ESI^+^ mode. ESI^+^ mode was finally used to detect RPDQ after both the ESI^+^ mode and the ESI^-^ mode being tested. Other parameters were also screened for ionization optimization. Full-scan product ion spectra of the [M + H]^+^ ions and the fragmentation pathways of RPDQ and IS are shown in Figure 1. Precursor ion and product ions were chosen according to the stability and ion response. The transition *m*/*z* 609.4348→143.1068 and 461.3941→127.1119 were chosen for the quantitation of RPDQ and IS, respectively.

#### 2.1.2. Method Validation

**Figure 2:**
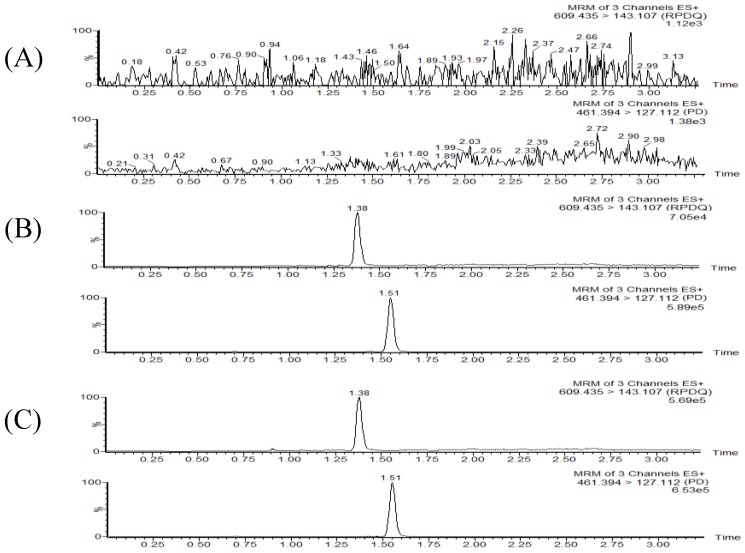
Representative chromatograms of (**A**) blank plasma, (**B**) blank plasma spiked with RPDQ and IS, and (**C**) a real plasma sample collected at an oral dose of 12 mg/kg RPDQ.

There was high selectivity in the detection of the RPDQ (RT, 1.38 min) and IS (RT, 1.51 min) by MRM without significant endogenous interference. Typical chromatograms of a blank plasma sample (A), a blank plasma sample spiked with the analytes at 300 ng/mL and IS at 100 ng/mL (B), and a plasma sample (RPDQ, 1000 ng/mL) collected at 6 h after an oral dose of 12 mg/kg RPDQ (C) are shown in Figure 2.

High linearity could be observed from the standard calibration curve ranging from 5 to 1000 ng/mL for RPDQ (*r*^2^ = 0.9966). The LLOQ was 5 ng/mL, which was already adequate for the detection of RPDQ in the pharmacokinetic study. The intra-day and inter-day precision was 1.51~3.51% and 2.23~5.41%, respectively. The intra-day and inter-day accuracy ranged from −5.61% to 2.87% and from −8.82 to 3.47%, respectively (Table 1). The variation of the IS measurements was less than 15%.

The best recovery of RPDQ was obtained by using protein-precipitating method. The recoveries of RPDQ at 15, 200, and 800 ng/mL were 92.90 ± 5.32, 85.66 ± 4.93, and 87.33 ± 1.68%, respectively. The recovery of the IS was 89.41 ± 5.21%. It was shown that the preparation efficiency of RPDQ and IS in the study was acceptable.

The matrix effect for RPDQ or IS was assessed by using QC plasma samples (15, 200, and 800 ng/mL) or using 20*R*-panaxadiol sample (80 ng/mL). The average matrix effect values were 85.11 ± 2.42, 93.06 ± 4.78, and 84.76 ± 3.68% for RPDQ at three QC concentrations, respectively. The matrix effect on IS was 95.15 ± 2.49% at the tested concentration.

The stability results (Table 2) showed that RPDQ was stable after it was placed at 25 °C for 4 h or at −20 °C for 14 days, or after experiencing three freeze–thaw (−20 to 25 °C) cycles. The results of diluted reliability are shown in Table 3, suggesting that the dilution did not affect the quantitative results of plasma samples.

#### 2.1.3. Pharmacokinetic Study

**Figure 3:**
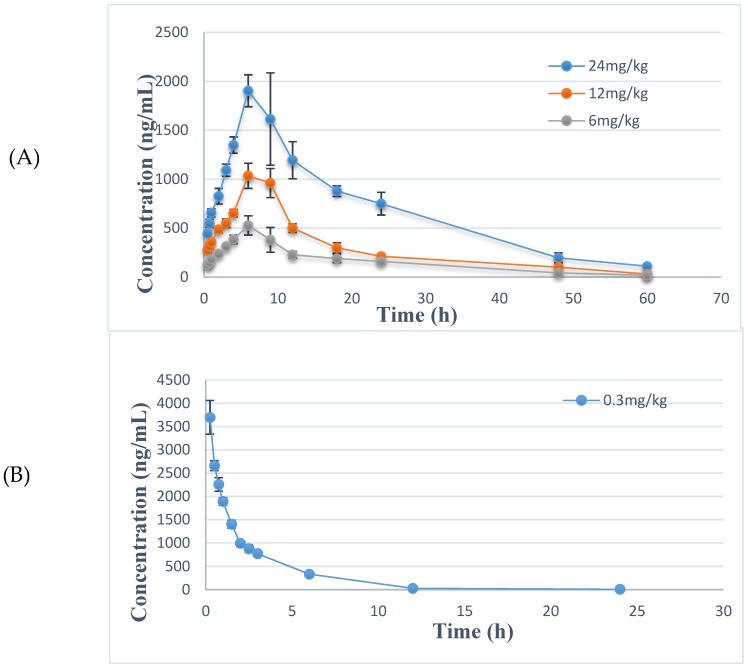
Mean plasma concentration–time profiles of RPDQ after intragastric administration (**A**) and intravenous administration (**B**) in rats.

The established method was then applied in a pharmacokinetic study of RPDQ after oral and intravenous administration in the present study. All of the data were calculated by the statistical software of DAS 3.0. (Shanghai Bojia Pharmatech Co.Ltd., Shanghai, China). From the results of concentration–time profiles (Figure 3) and the main pharmacokinetic parameters (Table 4), it was concluded that RPDQ was slowly cleared (*CL*, 0.04 ± 0.001 L/h/kg; *t*_1/2_, 2.94 h) and high extravascular distribution (*V*_d_, 11.52 ± 0.02 L/kg) after intravenous administration. On the other hand, RPDQ was detected at the first sample collection time (0.25 h) and reached the peak concentration at 7.25 h after oral administration. It was suggested that RPDQ was absorbed very slowly through the gastrointestinal tract. Moreover, the RPDQ exhibited slowly elimination (*t*_1/2_, 12.83 h) from plasma, and it could still be quantified in plasma at 60 h after gastrointestinal administration. As well as the low values of AUC, the mean of *F* was 5.15%. It was indicated that the analytical method applied to the pharmacokinetic study of RPDQ in rats was suitable and sufficient. In a word, the characteristic pharmacokinetic properties of RPDQ included slow oral absorption, low clearance, and poor absolute bioavailability.

#### 2.1.4. Results of Metabolites Identification of RPDQ

To determine the fragmentation patterns, the reference compound of RPDQ was used for the major MS/MS fragments in UPLC-QTOF-MS^E^. Just as shown in Table 5, RPDQ generated a protonated ion [M + H]^+^ at *m*/*z* 609.4346 and a number of fragment ions at *m*/*z* 549.3856, 477.3938, 459.3833, 441.3727, 423.3621, 381.3152, 283.2420, and 143.1067. The retention time of RPDQ was 16.64 min at the UPLC elution, and the molecular ion was *m*/*z* 609.4346 in mass spectrum.

**Figure 4:**
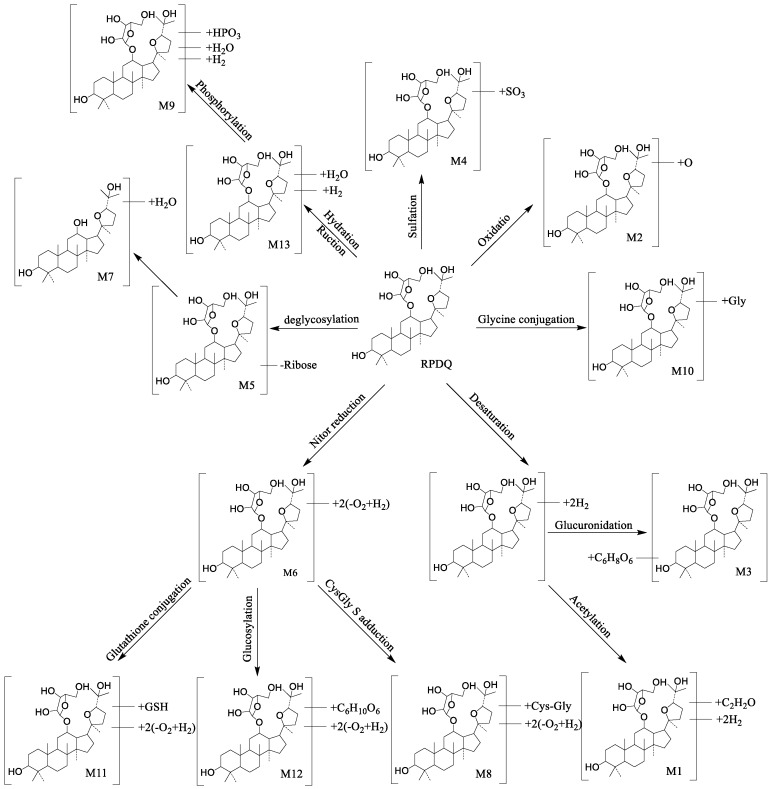
The proposed metabolic pathways of RPDQ in rats.

By using the UPLC-Q/TOF-MS method and being analyzed in parallel with blank control, RPDQ-dosed plasma, bile, urine, and feces samples were analyzed. In spite of lacking standards for the metabolites, the structures of them could be estimated based on retention time and mass spectral patterns between RPDQ and its product ions. According to certain rules, such as mass accuracy (±5 ppm), isotopic pattern, nitrogen rule, and double-bond equivalents, the most possible molecular formulas of metabolites were analyzed. Furthermore, based on the MS/MS fragmentation, the tentative chemical structures were identified and common metabolic pathways were profiled. There were 13 metabolites (including 4 Phase I metabolites and 9 Phase II metabolites were shown in Figure 4) whose structures were elucidated and summarized as follows.

*Phase I Metabolites*. **M1**, eluted at 13.09 min, was with the [M + H]^+^ ion at *m/z* 647.4144 (C_37_H_58_O_9_), 38 Da higher than that of RPDQ. This suggested that the metabolite was oxidation and acetylation metabolite. According to the characteristic product fragments at *m*/*z* 357.2788, *m*/*z* 355.2632, and *m*/*z* 439.3570, estimated binding sites at C-3 [24].

**M2** (RT = 13.10 min) showed a protonated ion at *m*/*z* 625.4330, suggesting that the molecular formula was C_35_H_60_O_9_. This metabolite was 16 Da higher than RPDQ. Compared with *m*/*z* 477.3938 and *m*/*z* 441.3727 from RPDQ, the diagnostic ion *m*/*z* 493.3866 and *m*/*z* 457.3649 was 16 Da higher than them. Therefore, **M2** was identified as an oxidrolysis metabolite of RPDQ [25].

**M6** was eluted at 25.20 min, showed an accurate protonated ion at *m*/*z* 549.4867 (C_35_H_64_O_4_), 60 Da less than that of RPDQ. This observation suggested that the metabolite was a reduction and oxidation metabolite. The characteristic fragment ion (*m*/*z* 416.4376) signified that reduction and oxidation occurred at the parent nucleus. The other main productions were *m*/*z* 541.3839, *m*/*z* 521.3837, and *m*/*z* 477.3575.

**M13** was eluted at 28.18 min, with [M + H]^+^ ion at *m*/*z* 629.4687(C_35_H_64_O_9_), 20 Da higher than that of RPDQ. This suggested that it was a reduction and hydration metabolite. It was implied that the reduction and hydration occurred at the parent nucleus [26], according to the characteristic fragment ion (*m*/*z* 569.4427).

*Phase II Metabolites.***M3**, eluted at 13.34 min, was with the [M + H]^+^ ion at *m*/*z* 781.4389 (C_40_H_64_O_14_), 172 Da higher than of RPDQ. This suggested that it was an oxidation and glucuronidation metabolite. The characteristic fragment ion at *m*/*z* 545.3475 meant that oxidation occurred at the parent nucleus. The characteristic fragment ion at *m*/*z* 723.4678 and *m*/*z* 315.2296 signified that glucuronidation occurred at C-20.

**M4** was eluted at 13.49 min, with the predominant quasi-molecular ion [M + H]^+^ *m*/*z* 689.3936 (C_35_H_60_O_11_S), 80 Da more than that of RPDQ, which implies that it was a sulfated product of RPDQ. The characteristic fragment ion at *m*/*z* 539.3401 and *m*/*z* 521.3295 signified that sulfation occurred at the parent nucleus. Therefore, **M4** was identified as the sulfation metabolite of RPDQ [26].

**M5** was eluted at 21.39 min, with the [M + H]^+^ ion at *m*/*z* 477.3947, 132 Da (ribose) lower than that of RPDQ. Moreover, the MS fragmentation patterns were similar to the reference substance of PDQ, with the characteristic fragment ion at *m*/*z* 441.3727, *m*/*z* 283.2420, and *m*/*z* 143.1067. Hence, **M5** was the aglycone of RPDQ.

**M7,** the metabolite at *m*/*z* 459.3815 (C_30_H_50_O_3_), 50 Da less than that of RPDQ, was eluted at 18.06 min. The characteristic ions included *m*/*z* 457.3675, *m*/*z* 441.3727, and *m*/*z* 439.3570. The product ions at *m*/*z* 457.3675 and *m*/*z* 439.3570 suggested that it was a deglycosylation and hydration metabolite. The characteristic fragment ion at *m*/*z* 441.3727 and *m*/*z* 143.2011 signified that deglycosylation occurred at C-12.

The molecular formula of **M8** and **M12** were C_40_H_72_N_2_O_7_S and C_41_H_72_O_10_, respectively, and they were 176 Da higher than that of **M6**. They were eluted at 25.65 and 27.91 min, showing the ions at *m*/*z* 725.5117 and *m*/*z* 725.5220, respectively. Furthermore, the distinctive fragment ions at *m*/*z* 549.4657, indicating that **M8** was the metabolite of cysgly S adduction. The typical fragment ion at *m*/*z* 143.1254 and 549.4657 signified that cysgly S adduction occurred at C-3. Distinctive fragment ions at *m*/*z* 549.4758 were observed, indicating that **M12** was a metabolite of glucosylation. The characteristic fragment ion at *m*/*z* 549.4758 and 143.1014 signified that glucosylation occurred at C-3 [27].

**M9** was eluted at 27.34 min and showed a predominant quasi-molecular ion [M + H]^+^ *m*/*z* 709.4263 (C_35_H_65_O_12_P), 80 Da more than that of RPDQ, suggesting that it was a reduction, hydration and phosphorylation product of RPDQ. The characteristic fragment ion at *m*/*z* 505.3441, *m*/*z* 481.3888, and *m*/*z* 463.3781 signified that phosphorylation occurred at the parent nucleus. Therefore, **M9** was identified as the phosphorylation metabolite of RPDQ [28].

**M10**, with the molecular formula C_37_H_63_NO_9_, which was 57 Da higher than that of RPDQ, were eluted at 27.35 min and showed the protonated ions at *m*/*z* 666.4598. The ions at *m*/*z* 495.3754 and *m*/*z* 516.3819 suggested that the metabolite was glycine conjugation metabolite. The characteristic fragment ion (*m*/*z* 495.3754) suggested that glycine conjugation occurred at the parent nucleus [29,30].

**M11**, C_45_H_79_N_3_O_9_S, suggesting 289 Da higher than that of **M6**. It was eluted at 27.65 min and showed the protonated ions at *m*/*z* 838.5641. The product ions at *m*/*z* 549.4627 and *m*/*z* 718.4459 suggested that the metabolite was glutathione conjugation metabolite. The fragment ion at *m*/*z* 549.4627, *m*/*z* 718.4459, and *m*/*z* 143.0924 signified that glutathione conjugation occurred at C-3 [29,30].

In summary, an analytical strategy for fast screening and identification metabolites of RPDQ by using UPLC-Q-TOF-MS^E^ and UNIFI 1.7.0 software (Waters, Manchester, UK) was explored in this study. According to 13 *metabolic* pathways, Phase II metabolites were the main metabolic pathway, including glycine conjugation, sulfation, phosphorylation, glucuronidation, glutathione, cysgly S conjugation, adduction, glucosylation and deglycosylation. Moreover, the Phase I metabolites included oxidation, acetylation, hydration, and reduction reactions. These pathways indicated that the hydroxy of RPDQ was the major metabolic site in vivo. The results provide a theoretical foundation and a better understanding of the biotransformation of RPDQ.

## 3. Discussion

Aiming at discussing the characteristics of the ginsenoside with glycosylation site at C-12, the pharmacokinetic and metabolism studies of RPDQ were carried out. Firstly, a UPLC-MS/MS method, with a short running time (3.5 min) and a high sensitivity (5 ng/mL), was validated and developed for the pharmacokinetic study. Secondly, pharmacokinetic analysis suggested that RPDQ was slowly absorbed, slowly eliminated, and had poor bioavailability in rats. Compared with other ginsenosides (Rh_1_, Rh_2_, and CK) also containing one glycosyl group, the elimination half-life (*t*_1/2_) takes much longer time and the bioavailability was higher than them. Meanwhile, compared with ginsenosides (Rb_1_, Rb_2_, and Rb_3_) containing two or more polysaccharide groups, the elimination half-life (*t*_1/2_) was lower, but the bioavailability was higher. On the other hand, compared with 3-position, 6-position, and 20-position glycosyl ginsenosides (Rh_1_, Rh_2_, Rg_2_, Rg_3_, and CK), the elimination half-life of RPDQ was much longer than Rh_2_ and Rg_3_. The bioavailability of RPDQ was also better than them all [31,32,33]. Compared with sapogenin (PPD, PPT, and PDQ), the elimination half-life of RPDQ longer than them. However, the bioavailability of PPD and PDQ was much higher than that of RPDQ. Finally, the main metabolites were characterized, and the proposed metabolic pathways of RPDQ were also profiled. The findings suggest that Phase II reactions are the principal metabolic pathway of RPDQ in vivo. Meanwhile, metabolite identification results proved that RPDQ mainly underwent some metabolism in the liver, since all metabolites could been found in the bile, urine, and feces samples. Taking the results obtained in this study into consideration, we concluded that the hepatic microsome might be the major cause for the poor oral bioavailability of RPDQ in rats. Moreover, RPDQ was also detected in plasma, urine, bile, and feces samples, which explains why RPDQ had a slow clearance rate in rats. In addition, except common metabolisms such as oxidation, glucuronidation, and deglycosylation, glutathione conjugation was found due to the epoxidized groups linked at C-20.

## 4. Materials and Methods

### 4.1. Chemicals and Ragents

**Figure 5:**
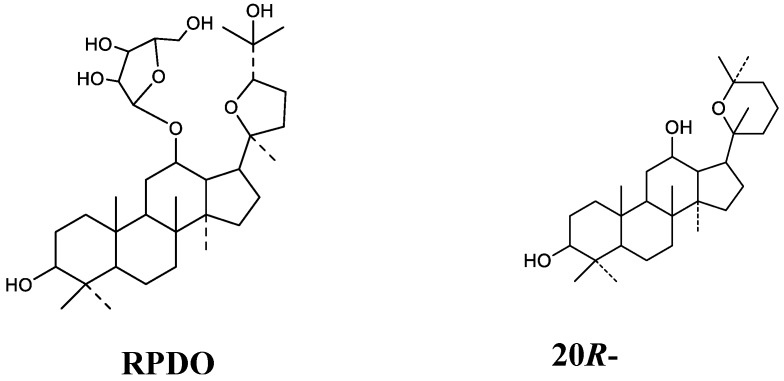
Chemical structures of 12-riboside-pseudoginsengenin DQ (RPDQ) and 20*R*-panaxadiol.

RPDQ, internal standard (IS, 20*R*-panaxadiol), and standard substance of **M5** (pseudoginsengenin DQ) with a purity of more than 98% were provided by the School of Pharmaceutical Sciences of Jilin University (Changchun, China). Their structures were confirmed using HR-MS and ^1^H and ^13^C-nuclear magnetic resonance (NMR) spectroscopy. The chemical structures of RPDQ and 20*R*-panaxadiol are shown in Figure 5. Acetonitrile and methanol used in the study were all UPLC-MS pure grade (Fisher Chemical Company, Geel, Belgium). Other chemicals were of analytical grade. Deionized water was purified using a Millipore water purification system (Millipore, Billerica, MA, USA). Formic acid was purchased from the Company of Sigma-Aldrich. Blank rat plasma used was prepared by our group.

### 4.2. Animals and Drug Administration

The experiments on animals were approved by the Review Committee of Animal Care and Use of Jilin University. The study was conducted according to the ethical principles for animal use and care. Wistar rats (200 ± 20 g) were provided by Changchun Yisi Laboratory Animal Co. Ltd. (Changchun, China). All rats were housed in an environmentally controlled room (12 h light/dark cycle; temperature: 22 ± 2 °C; relative humidity: 55 ± 5%), and except during fast before experiments, the rats were fed with standard laboratory food and water ad libitum.

In the pharmacokinetic study, the rats were divided into different groups (*n* = 6, three males and three females): (1) RPDQ (6 mg/kg, i.g.), (2) RPDQ (12 mg/kg, i.g.), (3) RPDQ (24 mg/kg, i.g.), and (4) RPDQ (0.3 mg/kg, i.v.).

For a metabolism study, RPDQ (48 mg/kg, i.g.) was administered to obtain a high concentration of metabolites.

### 4.3. Sample Preparation

#### 4.3.1. Pharmacokinetic Study

RPDQ and 20*R*-panaxadiol stock methanol solutions (1.00 mg/mL) were all prepared with the reference compounds, respectively. Firstly, blank rat plasma was prepared. The blood samples were collected immediately from the abdominal aorta and incubated at 37 °C for 30 min. The plasma was separated by centrifugation at 4000 rpm for 15 min, and was frozen at −20 °C until analysis. By spiking the dilute solutions with blank rat plasma, calibration curves were plotted to produce the points equivalent to 5, 10, 25, 50, 100, 300, 500, and 1000 ng/mL for RPDQ. With the same method as calibration standards, 15, 200, and 800 ng/mL of quality control (QC) samples were independently prepared. All the QC and calibration standards samples were prepared freshly before analysis.

From the fossa orbitalis vein, the whole blood (150 μL) was collected at 0.5, 0.75, 1, 2, 3, 4, 6, 9, 10, 12, 18, 24, 48, and 60 h after oral administration, and 0.25, 0.5, 0.75, 1, 1.5, 2, 2.5, 3, 6, 12, and 24 h after intravenous administration. All the blood samples were anticoagulated with sodium heparin. After collection, the whole blood was centrifuged at 4000 rpm and 4 °C for 20 min to obtain plasma.

Plasma samples (being stored at −20 °C before analysis) were thawed and vortexed at room temperature. The mixture of each plasma sample (50 μL) and 20*R*-panaxadiol solution (100 ng/mL, 500 μL) was vortexed (2 min) and centrifuged (13,000 rpm, 4 °C, 10 min). The clear supernatant was obtained and transferred into vials, and 2 μL of it were then kept in autosampler at 4 °C and injected for analysis.

#### 4.3.2. Metabolism Study

Blood samples were collected at 0.25, 0.5, 0.75, 1, 1.5, 2, 2.5, 3, 6, 12, and 24 h after intragastric administration. The pooled plasma sample (500 μL) was then added with methanol (2 mL) to vortex and precipitate proteins. Subsequently, the mixtures were centrifuged (10,000 rpm, 4 °C, 10 min) to obtain the supernatant, which was then evaporated by N_2_ to obtain the residue. Methanol (100 μL) was used to reconstitute the residue.

In order to obtain bile samples, rats were anaesthetized with urethane (1.0 g/kg, intraperitoneal injection). After making an abdominal incision, the bile was collecting by surgically inserting a plastic cannula into the bile ducts. Prior to administration, blank bile was collected for 2 h. After administration, bile samples were collected for 12 h.

By using metabolism cages, blank and dosed urine and feces samples were also collected prior to administration or 24 h after administration. After diluting each urine or bile sample (300 μL) with 1 mL of methanol, the mixture was centrifuged (10,000 rpm, 4 °C, 10 min) to obtain the supernatant, which was then evaporated by N_2_ to obtain the dried urine or bile residue. Methanol (50 μL) was then used to dissolve each residue. After centrifuging (10,000 rpm, 4 °C, 10 min), the clear supernatants were used for the analysis.

Each sample of dried feces powder (10 mg) was extracted with methanol (1 mL) by being ultrasonicated for 30 min in an ice-water bath. The mixture was then centrifuged (10,000 rpm, 4 °C, 10 min) to obtain the clear supernatant. Similarly, the supernatant was evaporated to obtain the dried residue, which was supplemented with 500 μL of methanol to be reconstituted and centrifuged at 10,000 rpm for 10 min at 4 °C. Additionally, all biological samples were stored at −20 °C.

### 4.4. Instruments and Experimental Conditions

#### 4.4.1. LC-MS/MS Conditions

The analysis of pharmacokinetic was conducted on a Waters ACQUITY UPLC System coupled with a XEVO TQ-S mass spectrometer equipped with an electrospray ionization source (Waters, Milford, MA, USA). Separation was performed on a Waters BEH C18 UPLC column (2.1 × 50 mm, 1.7 μm) at 40 °C. The mobile phase, 0.1% formic aqueous solution (A) and 0.1% formic acid in acetonitrile (B) in proportions, was adjusted with a gradient elution programme (0 min, 40% B; 0~2 min, 40~100% B; 2~2.5 min, 100% B; 2.5~2.6 min, 100~40% B; 2.6~3.5 min, 100% B). The flow rate was kept constant at 0.3 mL/min. Chromatography of the RPDQ and IS was performed within 3.5 min. The injection volume was 2 μL. The multiple reaction monitoring (MRM) mode was performed in ESI^+^. The main parameters were as follows: capillary voltage: 3000 V; source temperature: 150 °C; desolvation temperature: 300 °C; desolvation gas flow: 800 L/h; cone gas flow: 150 L/h; nebulizer gas flow: 7 bar; collision gas flow: 0.15 mL/min; cone voltage: 35 V and 28 V for RPDQ and IS, respectively; collision energy: 10 eV and 6 eV for RPDQ and IS, respectively. Masslynx V4.1 workstation (Waters, Milford, MA, USA) was used for data acquisition and processing [34,35,36,37,38].

#### 4.4.2. UPLC-QTOF/MS Conditions

A Waters ACQUITY UPLC System coupled with a Xevo G2-S Q-TOF mass spectrometer (Waters, USA) was used to profile the metabolites. An ESI source in the positive ionization mode was equipped. A Waters UPLC BEH C_18_ column (2.1 × 50 mm, 1.7 μm) was used with a column temperature at 30 °C and a flow rate at 0.4 mL/min. The autosampler temperature was set at 4 °C and the injection volume was 10 μL. The mobile phase was composed of 0.1% formic aqueous solution (A) and 0.1% formic acid in acetonitrile (B). The gradient elution programme was as follows: 0~2 min, 10% B; 2~26 min, 10→100% B; 26~28 min, 100% B; 28~28.1 min, 100→10% B, 28.1~30 min, 10% B. The following optimized conditions were employed: capillary voltage: 2.6 kV; cone voltage: 40 V; desolvation temperature: 300 °C; source temperature: 120 °C; cone gas flow: 50 L/h; desolvation gas flow: 800 L/h. The energy of low energy function and the collision energy of high energy function was set at 6 V and 20~40 V, respectively, in MS^E^ mode. The mass spectrometer was calibrated with sodium formate in a range of 100–1600 Da. Leucine-enkephalin (*m*/*z* 556.2771) was used as the lockmass at a flow rate of 10 μL/min and a concentration of 200 ng/mL. Data were collected by Masslynx V4.1 workstation in continuum mode. Metabolic characterization of RPDQ was analyzed using UNIFI 1.7.0 software (Waters, Manchester, UK) [39].

### 4.5. Method Validation

The specificity, linearity, LLOQ, precision and accuracy, extraction recovery, matrix effect, diluted reliability, and stability of the method were validated by following a non-clinical drug pharmacokinetic study technical guideline (China Food And Drug Administration 2014) and the Bioanalytical Method Validation Guideline (Chinese Pharmacopoeia 2015, Vol. 4). Specificity was assessed by comparing blank plasma samples, plasma was spiked with RPDQ and 20*R*-panaxadiol, and plasma samples were collected after administration. Linearity was analyzed through weighted regression (1/*x*^2^) of peak area ratios (*y*) of RPDQ to 20*R*-panaxadiol versus nominal concentration (*x*) in plasma. LLOQ was the lowest concentration on the calibration curve, with acceptable precision (RSD ≤ 20%) and accuracy (RE ≤ ± 20%), while precision and accuracy were assessed with QC samples (15, 200, and 800 ng/mL). The intra- and inter-day precision and accuracy were evaluated with the analysis of six replicate QC samples on three consecutive days, respectively. The extraction recovery and matrix effect at three QC concentrations were assayed. By comparing peak areas of RPDQ and 20*R*-panaxadiol in extracted QC samples with those in post-extracted blank plasma samples, the recoveries were calculated. Moreover, by comparing the peak area of samples spiked after extraction with that of the neat solution’s equivalent concentration, the matrix effect was then evaluated. Stability was evaluated by analyzing replicate QC samples, which were stored at −20 °C for two weeks at room temperature for 4 h, and experienced three freeze/thaw cycles (from −20 °C to room temperature) on consecutive days. The stabilities of RPDQ in stock solutions at 15 °C and stored in plastic autosampler vials for 16 h at 10 °C were also assessed. Five-fold higher concentrations of QC were achieved by adding moderate RPDQ to blank plasma. The samples were then diluted and analyzed with undiluted calibration standards. The accuracy and precision were within acceptable limits (± 15%).

### 4.6. Pharmacokinetic Study

Samples containing RPDQ above the upper limit of quantification were diluted and re-analyzed. The pharmacokinetics analysis in non-compartmental mode was conducted by Drug and Statistics (DAS) 3.0 pharmacokinetic software programme (Mathematical Pharmacology Professional Committee of China, Shanghai, China). The concentration versus the time curve was plotted. The time to reach the peak concentration (*T*_max_) and the maximum plasma concentration (*C*_max_) were obtained after oral administration. The results are shown as the mean ± standard deviation. The absolute bioavailability (*F*%) was calculated using the equation (AUCp.o. × Dosei.v.)/(AUCi.v. × Dosep.o.) × 100%.

## 5. Conclusions

In this study, the pharmacokinetic and metabolism studies of 12-riboside-pseudoginsengenin DQ (RPDQ) were performed for the first time in this study. In pharmacokinetic analysis, a UPLC-MS/MS method for quantification was developed and validated. The method was then successfully applied to the pharmacokinetic study after intravenous administration of 0.3 mg/kg RPDQ and intragastric administration of 6, 12, and 24 mg/kg RPDQ. The results showed that RPDQ exhibited slow oral absorption (*T*_max_ = 7.5 h), low elimination (*t*_1/2_ = 12.83 h), and poor absolute bioavailability (*F* = 5.15%). A metabolic investigation of RPDQ was carried out through UPLC-QTOF-MS^E^. The main metabolites and metabolic pathways were all characterized. Overall, compared with the ginsenosides with C-3, C-6, and C-20 glycoside positions, RPDQ has special pharmacokinetic parameters and metabolic pathways. These findings could provide data and reference for further research and applications of RPDQ.

## Figures and Tables

**Table 1:** Intra-day and inter-day precision and accuracy of RPDQ in rat plasma.

Concentration (ng/mL)	Inter-Day Measured Concentration (ng/mL)	Precision (RSD, %)	Accuracy (RE, %)	Intra-Day Measured Concentration (ng/mL)	Precision (RSD, %)	Accuracy (RE, %)
15	15.52 ± 0.84	5.41	3.47	15.43 ± 0.55	3.51	2.87
200	182.41 ± 6.87	3.48	−8.82	188.79 ± 3.56	1.79	−5.61
800	775.02 ± 17.75	2.23	−3.12	788.99 ± 12.04	1.51	−1.3

**Table 2:** The stability of RPDQ in rat plasma (*n =* 6).

	Concentration		Mean	Accuracy
	(ng /mL)		(ng /mL)	(%)
Short-term stability (25 °C, 4 h)	RPDQ	15	14.85 ± 0.72	−1.00
		200	188.12 ± 14.00	−5.94
		800	773.99 ± 26.01	−3.25
	IS	100	92.34 ± 3.12	−7.66
Long-term stability (10°C, 16 h)	RPDQ	15	15.33 ± 1.61	2.2
		200	186.96 ± 13.41	−6.52
		800	816.23 ± 15.82	2.03
	IS	100	91.34 ± 2.31	−8.66
Freezing stability (−20 °C, 14 d)	RPDQ	15	15.41 ± 1.47	2.74
		200	179.07 ± 14.73	−10.47
		800	786.32 ± 19.88	−1.71
	IS	100	94.74 ± 4.25	−5.26
Freeze–thaw stability (freeze–thaw cycles)	RPDQ	15	14.96 ± 0.58	−0.03
		200	188.7 ± 16.57	−5.65
		800	785.21 ± 19.19	−1.85
	IS	100	94.11 ± 3.47	−5.89

**Table 3:** The diluted reliability of RPDQ in plasma (*n* = 6).

Concentration after Dilution (ng/mL)	Mean (ng/mL)	Accuracy (%)	RSD (%)
15	14.82 ± 0.61	−1.20	4.11
200	187.79 ±12.19	−6.11	6.49
800	784.17 ± 21.17	−1.98	2.70

**Table 4:** Pharmacokinetic parameters after intragastric or intravenous administration in rats.

Dose (mg/kg)	*t*_1/2_ (h)	*T*_max_ (h)	AUC_(0__–__60)_ (ug/L/h)	AUC_(__0–__∞)_ (ug/L/h)	F (%)	Vz (L/kg)	CLz (L/h/kg)
6	12.59 ± 1.28	7.0 ± 1.55	9212.53 ± 608.41	9568.25 ± 545.64	5.55%	11.45 ± 1.55	0.63 ± 0.036
12	12.83 ± 0.56	7.5 ± 1.63	17,108.67 ± 987.76	17,685.11 ± 985.93	5.15%	12.53 ± 0.59	0.68 ± 0.022
24	13.74 ± 1.11	7.0 ± 1.55	40,358.37 ± 3441.44	42,553.53 ± 3207.59	6.08%	11.28 ± 1.69	0.57 ± 0.044
0.3 (i.v)	2.94 ± 0.51	0.25	8298.18 ± 258.5.	8325.73 ± 270.09	--	11.52 ± 0.02	0.04 ± 0.001

**Table 5:** Metabolites of RPDQ characterized by using UPLC-Q-TOF-MS^E^.

No.	RT (min)	Formula	Measured Mass (m/z)	Calculate Mass (m/z)	Error (ppm)	Fragment Ions	Metabolic Pathway	Source
RPDQ	16.65	C_35_H_60_O_8_	609.4346	609.8540	1.3	549.3856, 477.3938, 459.3833, 441.3727, 381.3152, 283.2420, 143.1067	parent	P,B, F,U
M1	13.09	C_37_H_58_O_9_	647.4144	647.8590	−1.4	475.3782, 457.3675, 439.3569, 421.3464, 357.2788, 355.2632, 143.1067	Desaturation Acetylation	F
M2	13.10	C_35_H_60_O_9_	625.4330	625.8534	3.3	493.3866, 457.3649, 475.3781, 439.3565, 143.1024	Oxidation	F,U
M3	13.34	C_41_H_64_O_14_	781.4389	781.9464	2.7	723.4678, 573.4149, 545.3472, 315.2318	Desaturation Glucuronidation	U
M4	13.49	C_35_H_60_O_11_S	689.3936	689.9172	1.1	675.3792, 670.3783, 539.3401, 521.3295, 503.3189, 441.3708, 143.1067	Sulfation	F
M5	21.39	C_30_H_52_O_4_	477.3932	477.7394	1.4	459.3832, 441.3727, 423.3621, 381.3152, 283.2420, 143.1067	Deglycosylation	P,B, F,U
M6	25.20	C_35_H_64_O_4_	549.4867	549.8882	−1.8	541.3839, 521.3837, 477.3575, 416.4376, 283.2457	Nitor reduction	F,U
M7	18.06	C_30_H_50_O_3_	459.3815	459.7241	4.7	457.3675, 441.3727, 439.3570, 421.3465, 45.3515, 381.3117, 143.1067	Desaturation Hydration	P,B, F,U
M8	25.65	C_40_H_72_N_2_O_7_S	725.5117	726.0818	−2.2	715.4351, 557.4126, 465.3574, 382.3957, 340.3538, 143.1254	CysGly S adduction	P,F
M9	27.34	C_35_H_65_O_12_P	709.4263	709.8651	1.9	505.3441, 495.4044, 481.3888, 463.3781	Phosphorylation	P,F,U
M10	27.35	C_37_H_63_NO_9_	666.4598	666.9054	3.2	551.39423, 534.4177, 516.3819, 495.3707, 143.1052	Glycine conjugation	P,B
M11	27.65	C_45_H_79_N_3_O_9_S	838.5641	839.1964	3.8	718.4459, 549.4627, 520.3758, 513.35745, 270.3118, 143.0924	Glutathione conjugation	P,B, F,U
M12	27.91	C_41_H_72_O_10_	725.5152	725.0123	3.1	557.3817, 549.4758, 443.3841, 143.1014	Glucosylation	P,B, F,U
M13	28.18	C_35_H_64_O_9_	629.4687	629.8852	1.0	594.4491, 569.4427, 461.3625, 303.3046, 143.2017	Reduction Hydration	B

## Supplementary Materials

The following are available online at http://www.mdpi.com/1420-3049/23/10/2499/s1: Structural determination of PPDQ. s2: Antitumor activity test of RPDQ in vitro by using S180, SPC-A-1 and A549 cells. s3: The representative spectra for the metabolites identification.
