# Supplementary material for: Pharmacokinetic and Metabolism Studies of 12-Riboside-Pseudoginsengenin DQ by UPLC-MS/MS and UPLC-QTOF-MSE
Source: Molecules. 2018 Sep 29;23(10):2499. doi: 10.3390/molecules23102499 (PMC6222672; doi:10.3390/molecules23102499)
Supplement: Supplementary file 1 [file molecules-23-02499-s001.pdf]

Supplementary Material 1

Structural determination of PPDQ

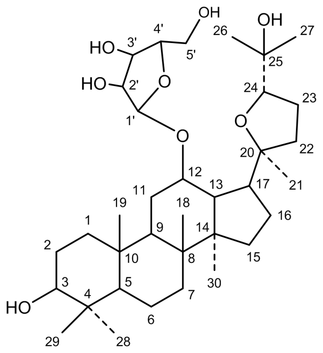

Compound was obtained as white power. The molecular formula of $C_{35}H_{60}O_8$ was determined by HR-ESI-MS at *m/z* 609.4346 $[M+H]^+$ (calcd for 609.4348) (Figure S1). According to the ^{1}H NMR (Figure S2), ^{13}C NMR (Figure S3), HMQC (Figure S4), HMBC (Figure S5), ^{1}H-^{1}H COSY and NOESY (Figure S7) spectra, the carbon and hydrogen signals of the compound were fully assigned. And the data were shown in Table S1.

Table S1. ^{1}H NMR (500MHz, pyridine-d5) and ^{13}C NMR (125.8MHz, pyridine-d5) data of RPDQ

No.	^{13}C NMR (δ)	^{1}H NMR (δ)	HMBC
1	39.30	1.27(1H, m)	C-2, 3, 5, 9, 10, 19
		0.71(1H, m)	C-2, 3, 5, 9, 10, 19
2	28.40	1.26(1H, m)	C-1, 3, 4
3	78.37	3.40(1H, m)	C-1, 4, 28, 29
4	39.90	-	-
5	56.79	0.76(1H, m)	C-3, 6, 9, 10, 19, 28
6	19.11	1.57(1H, m)	C-5
		1.44(1H, m)	C-4, 5
7	35.50	1.46(1H, m)	C-5, 8, 10, 14, 18
		1.27(1H, m)	C-5, 8, 9, 11
8	40.32	-	-
9	50.97	1.436(1H, m)	C-1, 5, 12, 14, 18
10	37.79	-	-
11	33.10	2.12(1H, m)	C-8, 9, 12
		1.43(1H, m)	C-5, 10, 18
12	76.54	4.14 (1H, d, J = 6.1 Hz)	C-13, 14, 1'
13	49.48	2.48(1H, m)	C-8, 11, 12, 15, 16, 17, 20
14	53.11	-	-
15	32.68	1.61(1H, m)	C-14, 16, 30
		1.06(1H, m)	C-8, 9, 11, 30
16	28.4	2.00(1H, m)	-
		1.96(1H, m)	C-8, 13,12, 14, 15, 17, 20, 30
17	49.12	1.93(1H, m)	C-12, 13, 14, 15, 20, 30
18	16.07	0.95(3H, s)	-
19	16.85	0.82(3H, s)	C-1, 5, 9, 10
20	87.27	-	-
21	26.77	1.36(3H, s)	C-22
22	32.60	2.00(1H, m)	C-17, 20, 27
		1.74(1H, m)	C-23, 24, 27
23	28.45	2.17(1H, m)	C-22, 24, 25
		1.75(1H, m)	C-22, 24
24	86.06	3.95(1H, m, H-24)	C-26, 27
25	71.13	-	-
26	26.96	1.33(3H, s)	C-24, 25, 20, 22, 21, 17
27	29.00	1.21(3H, s)	C-24, 25, 26,
28	28.50	1.26(3H, s)	C-3, 4, 5, 29
29	16.65	1.03(3H, s)	C-3, 4, 5, 28
30	18.75	0.97(3H, s)	C-8, 13, 14, 15
1'	98.49	5.47 (1H, d, J = 7.0 Hz)	C-12, 5', 3' 2'
2'	72.52	4.03(1H, m)	C-1', 5'
3'	72.23	4.73(1H, m)	C-1', 2', 4', 5'
4'	69.50	4.28(1H, m)	C-1', 2', 4', 5'
5'	65.48	4.27 (1H, d, J = 9.0 Hz)	C-1', 2', 4', 5'
		4.17 (1H, d, J = 5.8 Hz)	C-1', 2', 4', 5'

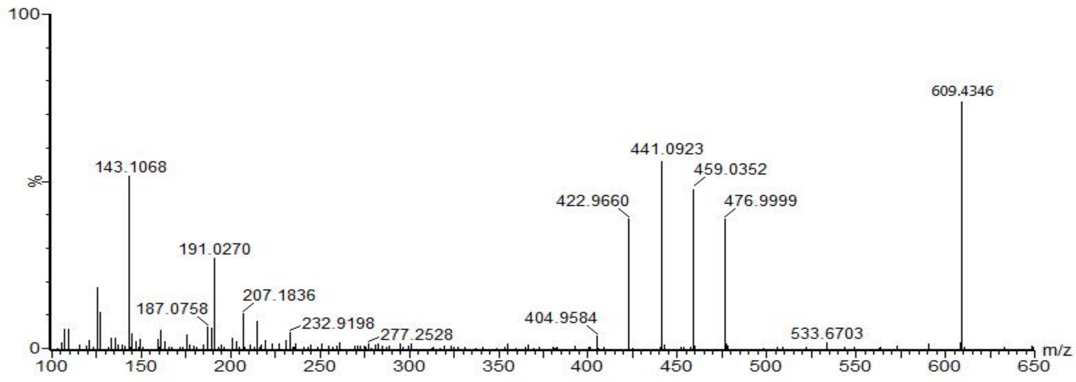

Figure S1. HR-ESI-MS spectrum of RPDQ

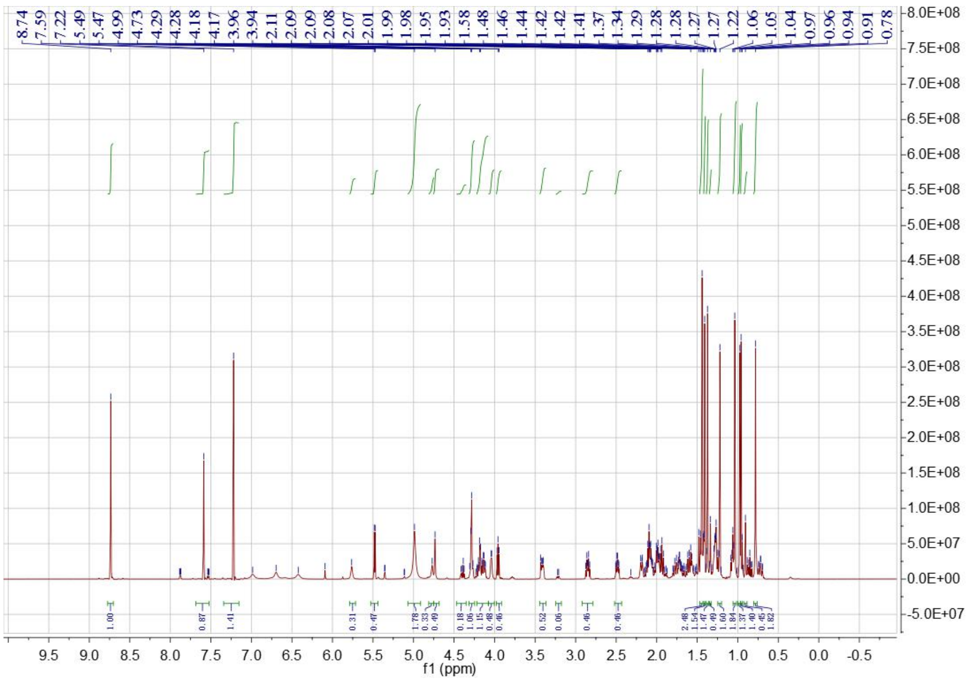

Figure S2. ^{1}H NMR spectrum of RPDQ

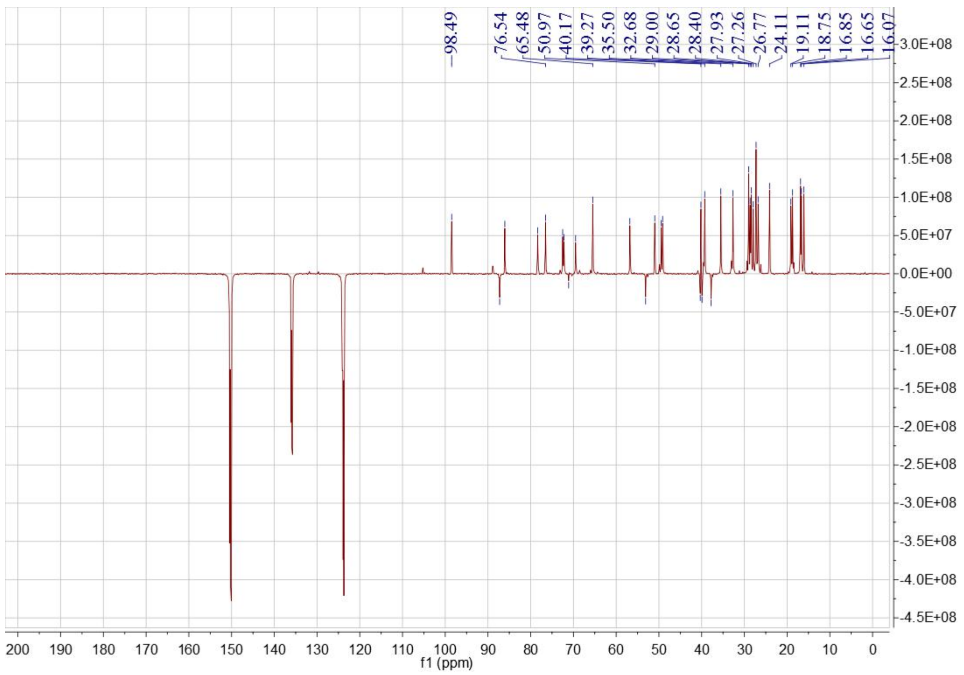

Figure S3. ^{13}C NMR (DEPTQSP) spectrum of RPDQ

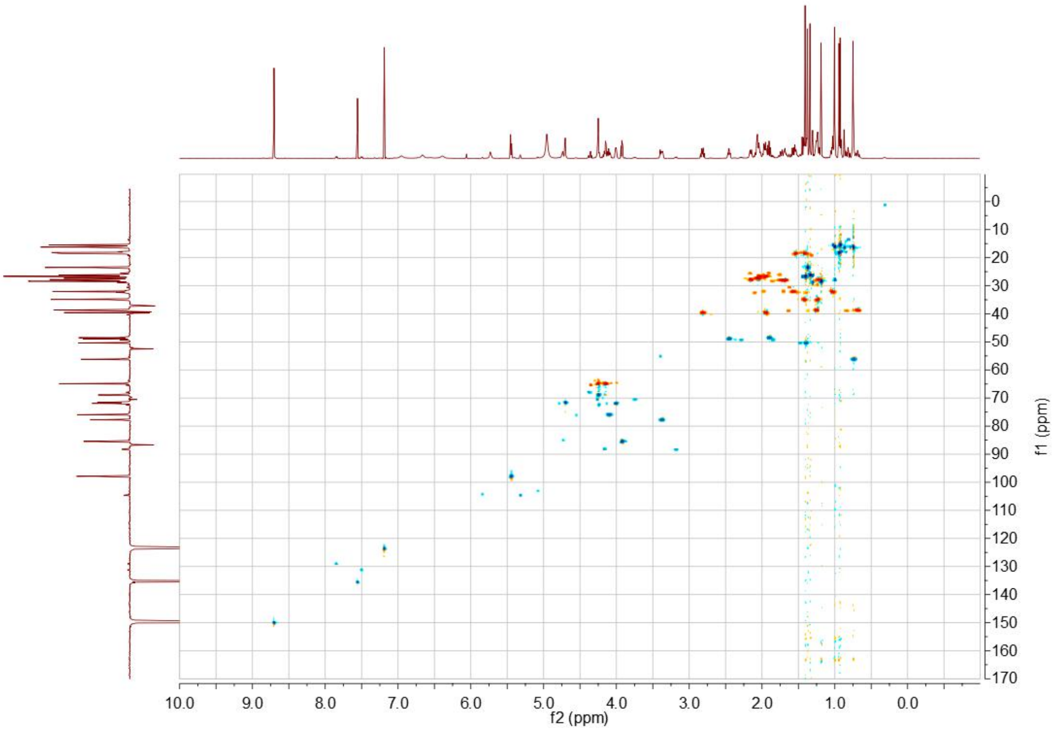

Figure S4. HMBC-related signals of RPDQ

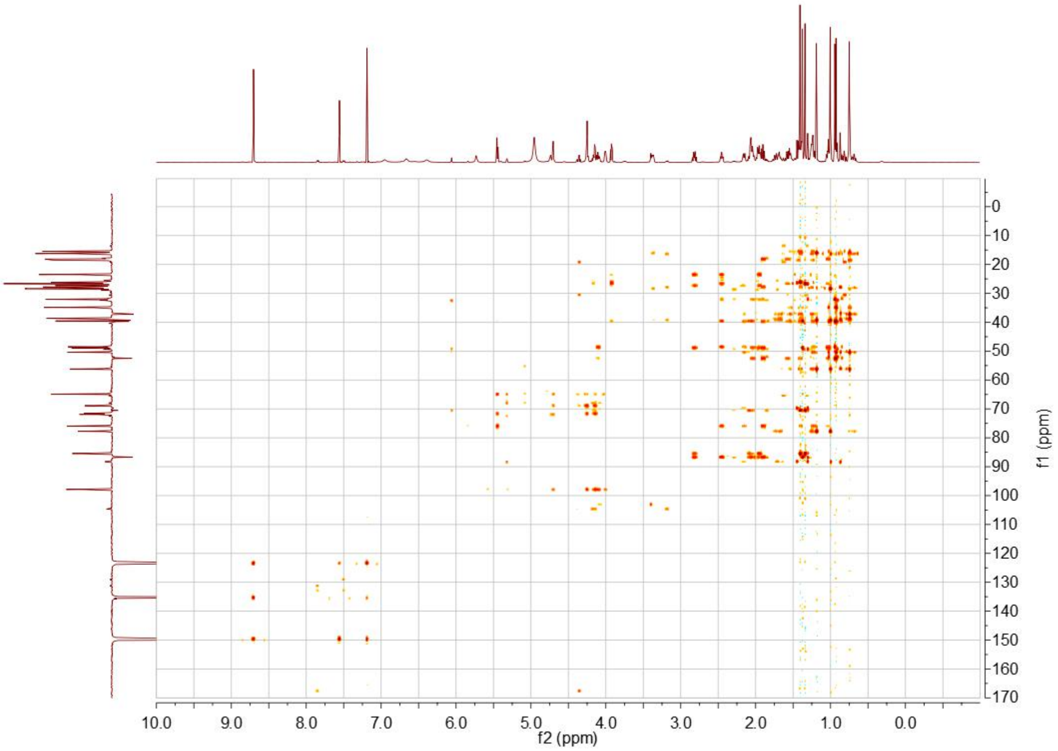

Figure S5. HMQC spectrum of RPDQ

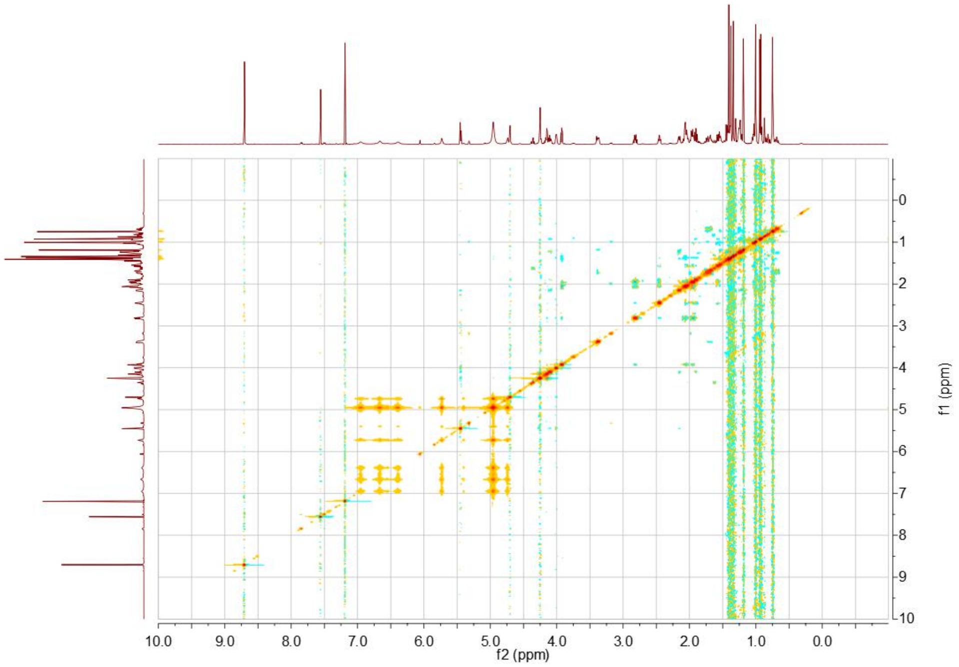

Figure S6. ^{1}H-^{1}H COSY spectrum of RPDQ

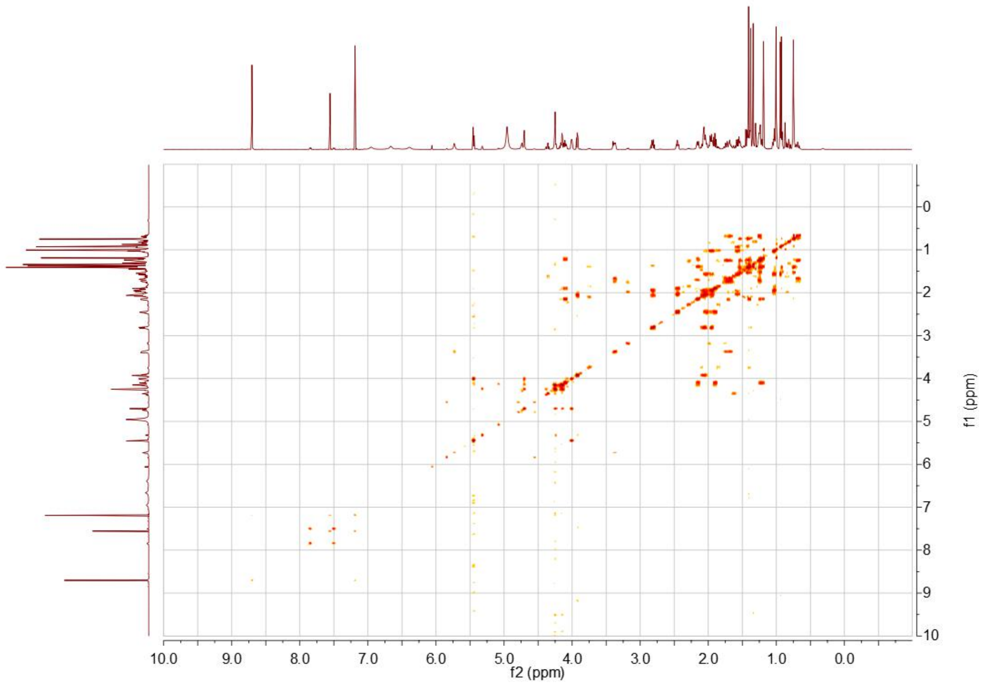

Figure S7. NOESY spectrum of RPDQ

Supplementary Material 2

Antitumor activity test of RPDQ *in vitro*

by using S180, SPC-A-1 and A549 cells

Since the start material for the semi-synthesis of 12-Riboside-pseudoginsengenin DQ (RPDQ) was pseudoginsengenin DQ (PDQ), the antitumor activities of RPDQ and PDQ were both tested. The results showed that RPDQ had a better antitumor effect than PDQ.

1. Tumor cells

The SPC-A-1 and S180 tumor cell lines were purchased from Wuhan Procell Life Science & Technology Co,.Ltd. A549 tumor cell lines were purchased from iCell Bioscience Inc (Shanghai) Biological Co., Ltd.

2. Experimental procedure

2.1 S180 cells

S180 cells (1 × 105 cells/mL) were seeded into a 96-well plate. After 24 h, pretreated with various concentrations of RPDQ or PDQ (1-200 μM) for 44 h at 37℃, and the cells were treated with MTT for 4 h. The formazan crystal product in the cells were dissolved with acid isopropanol. The absorbance was measured at 570 nm with a microplate reader.

2.2 SPC-A-1/A549 cells

SPC-A-1/A549 cells (1 × 105 cells/mL) were seeded into a 96-well plate. After 24 h, pretreated with various concentrations of RPDQ or PDQ (1-200 μM) for 44 h at 37℃, and the cells were treated with MTT for 4 h. The formazan crystal product in the cells were dissolved with DMSO. The absorbance was measured at 490 nm with a microplate reader.

2.3 Cell viability

Cell viability was calculated by the following formula: Cell viability (%)= $1-\{(OD_{control\ group}-OD_{treated\ group})/OD_{control\ group}\times 100\%\}$.

2.4 Statistical analyses

All data were processed by statistical software SPSS 17.0. The data were expressed as Mean ± Standard Deviation. The differences between groups were analyzed by one-way analysis of variance. The *t*-test was used for comparison between groups. $P<0.05$ was considered statistically significant.

3. Results

The lower cell viability suggested the better anti-proliferation effect on tumor cell. The assay of cell viability of RPDQ and PDQ treated *in vitro* were shown in Figure **S1-S3**. It could be concluded that RPDQ had good anti-proliferation effects on S180 cells, SPC-A-1 cells and A549 cells. Moreover, RPDQ showed a better anti-tumor effect than PDQ.

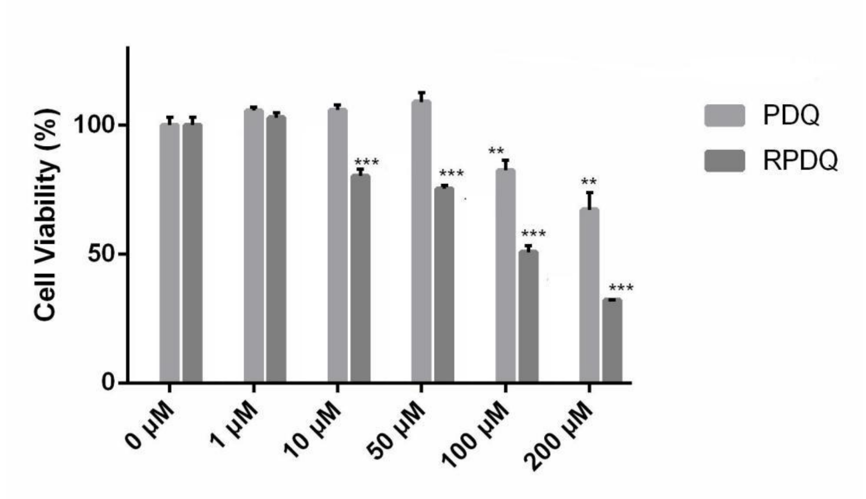

Figure S1: Effects of PDQ and RPDQ on the proliferation of S180 cells

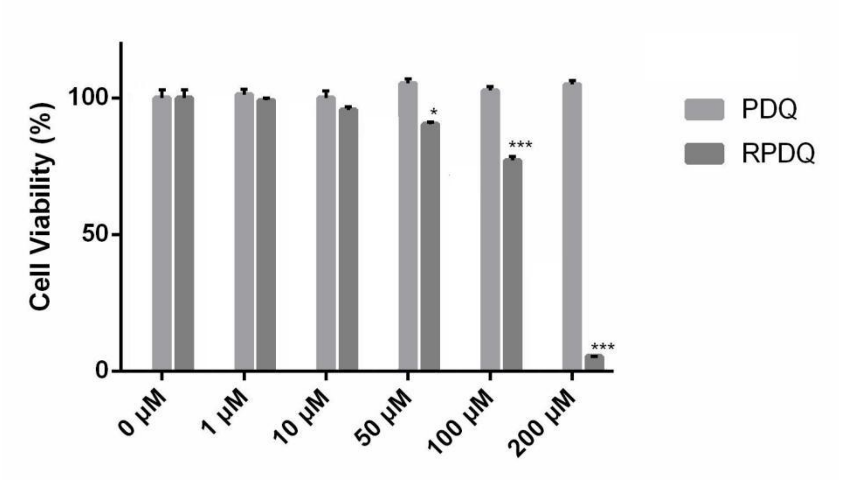

Figure S2: Effect of PDQ and RPDQ on the proliferation of SPC-A-1 cells.

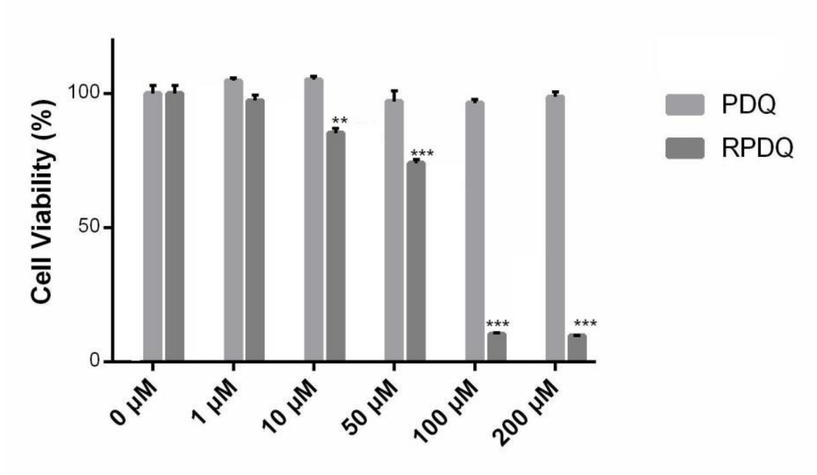

Figure S3: Effect of PDQ and RPDFQ on the proliferation of A549 cells.

Supplementary Material 3

The representative spectra for the metabolites identification

M1

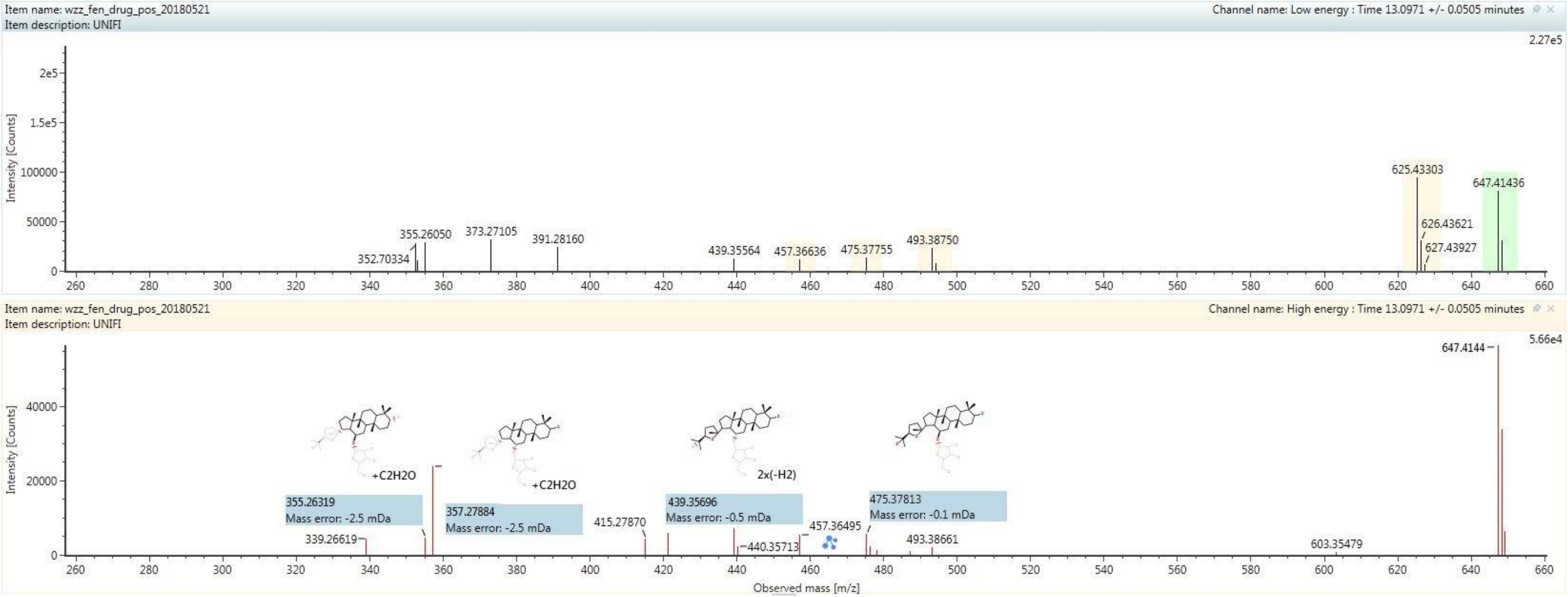

M2

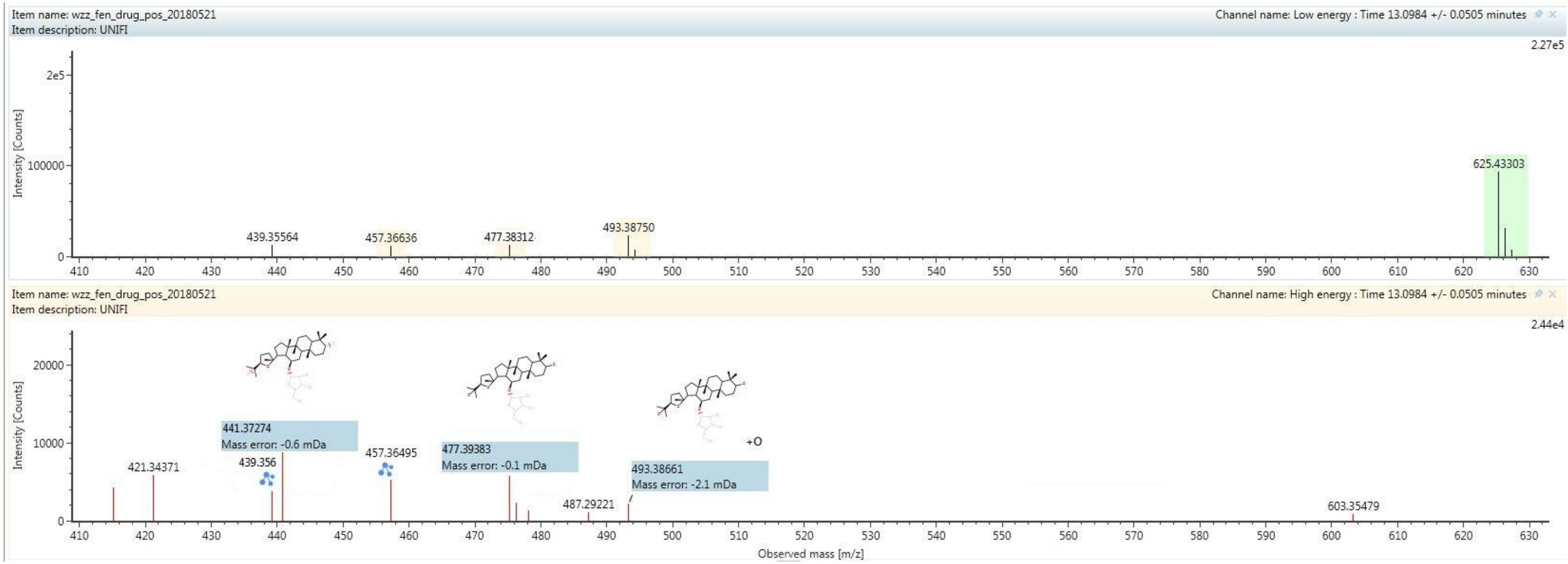

Item name: wzz_fen_drug_pos_20180521
Item description: UNIFI
Channel name: Low energy : Time 13.0984 +/- 0.0505 minutes
2.27e5
Intensity [Counts]
625.43303
439.35564
457.36636
477.38312
493.38750
Item name: wzz_fen_drug_pos_20180521
Item description: UNIFI
Channel name: High energy : Time 13.0984 +/- 0.0505 minutes
2.44e4
441.37274
Mass error: -0.6 mDa
439.356
421.34371
457.36495
477.39383
Mass error: -0.1 mDa
493.38661
Mass error: -2.1 mDa
+O
487.29221
603.35479
Observed mass [m/z]

M3

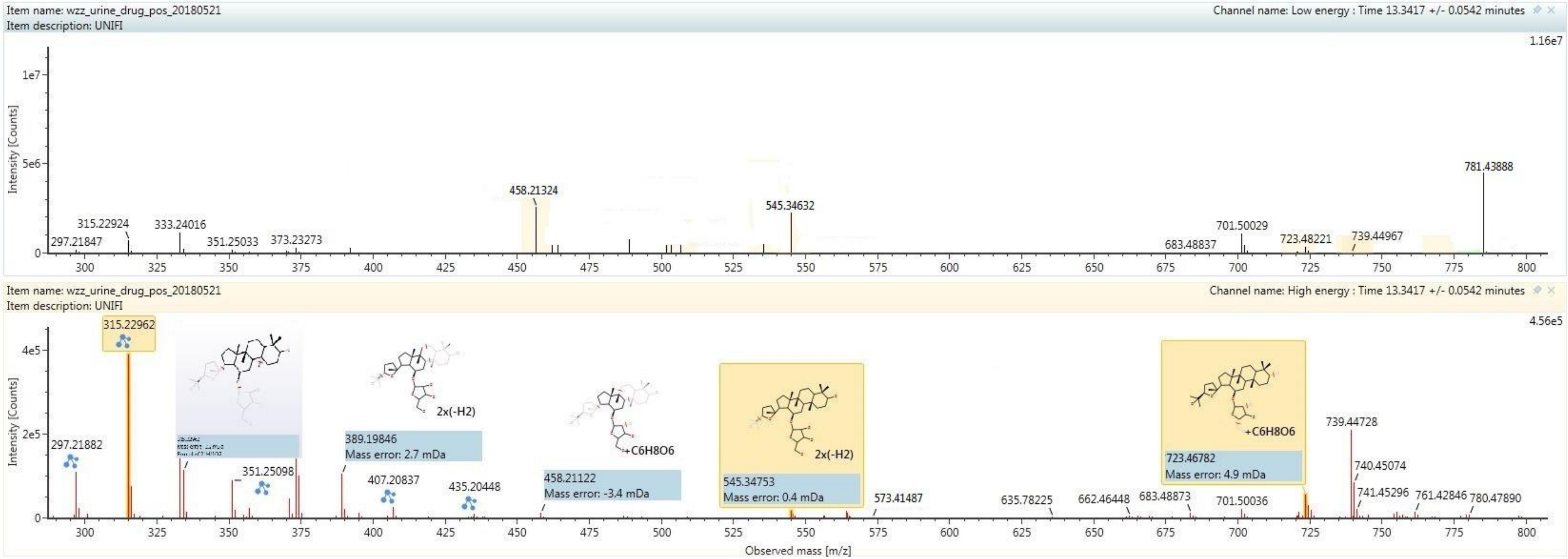
Item name: wzz_urine_drug_pos_20180521
Item description: UNIFI
Channel name: Low energy : Time 13.3417 +/- 0.0542 minutes
1.16e7
Intensity [Counts]
297.21847
315.22924
333.24016
351.25033
373.23273
458.21324
545.34632
683.48837
701.50029
723.48221
739.44967
781.43888
Item name: wzz_urine_drug_pos_20180521
Item description: UNIFI
Channel name: High energy : Time 13.3417 +/- 0.0542 minutes
4.56e5
297.21882
315.22962
351.25098
389.19846
Mass error: 2.7 mDa
2x(-H2)
407.20837
435.20448
458.21122
Mass error: -3.4 mDa
+C6H8O6
545.34753
Mass error: 0.4 mDa
2x(-H2)
573.41487
635.78225
662.46448
683.48873
701.50036
723.46782
Mass error: 4.9 mDa
+C6H8O6
739.44728
740.45074
741.45296
761.42846
780.47890
Observed mass [m/z]

M4

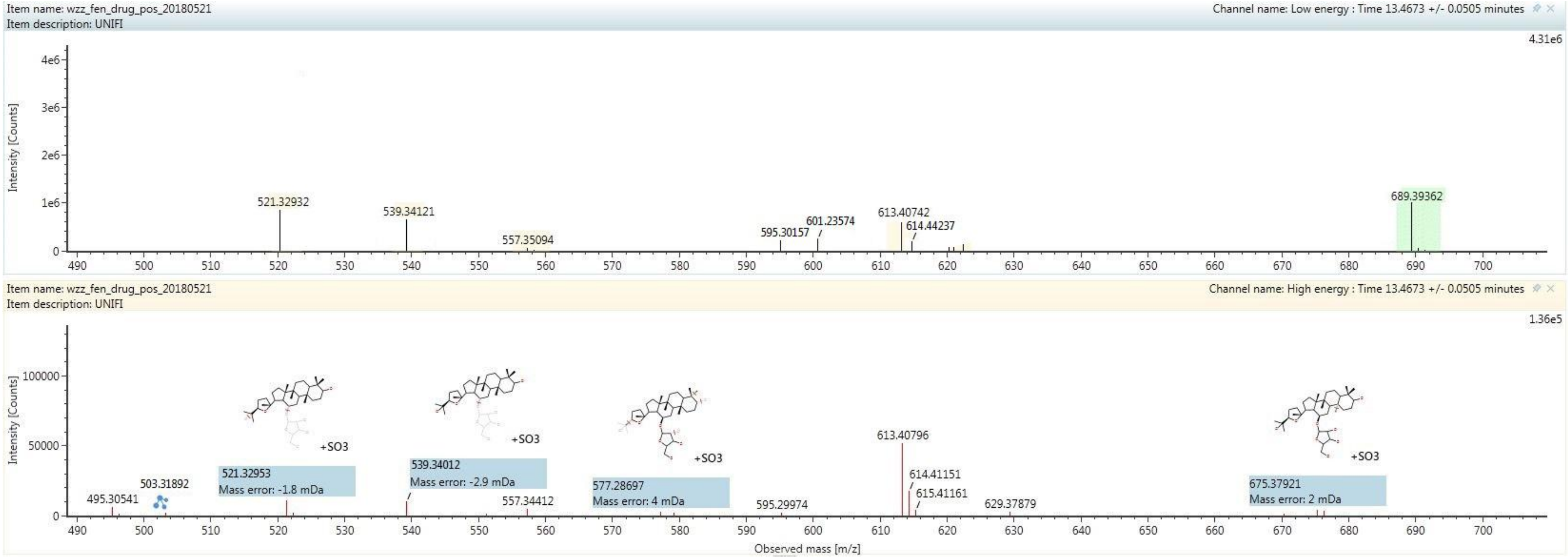

Item name: wzz_fen_drug_pos_20180521
Item description: UNIFI
Channel name: Low energy : Time 13.4673 +/- 0.0505 minutes
4.31e6
Intensity [Counts]
521.32932
539.34121
557.35094
595.30157
601.23574
613.40742
614.44237
689.39362
Item name: wzz_fen_drug_pos_20180521
Item description: UNIFI
Channel name: High energy : Time 13.4673 +/- 0.0505 minutes
1.36e5
Intensity [Counts]
495.30541
503.31892
521.32953
Mass error: -1.8 mDa
+SO3
539.34012
Mass error: -2.9 mDa
557.34412
577.28697
Mass error: 4 mDa
595.29974
613.40796
614.41151
615.41161
629.37879
675.37921
Mass error: 2 mDa
Observed mass [m/z]

M5

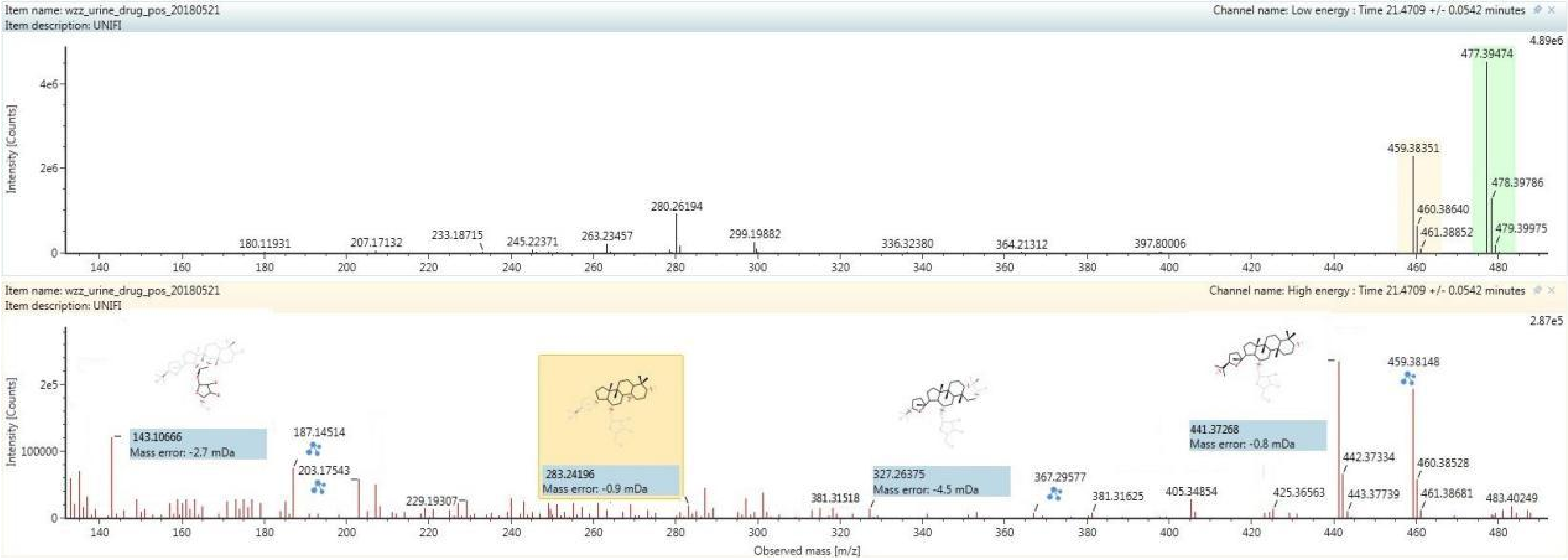
Item name: wzz_urine_drug_pos_20180521
Item description: UNIFI
Channel name: Low energy : Time 21.4709 +/- 0.0542 minutes
4.89e6
Intensity [Counts]
477.39474
459.38351
478.39786
460.38640
461.38852
479.39975
280.26194
180.11931
207.17132
233.18715
245.22371
263.23457
299.19882
336.32380
364.21312
397.80006
Item name: wzz_urine_drug_pos_20180521
Item description: UNIFI
Channel name: High energy : Time 21.4709 +/- 0.0542 minutes
2.87e5
143.10666
Mass error: -2.7 mDa
187.14514
203.17543
229.19307
283.24196
Mass error: -0.9 mDa
327.26375
Mass error: -4.5 mDa
367.29577
381.31518
381.31625
405.34854
425.36563
441.37268
Mass error: -0.8 mDa
442.37334
443.37739
459.38148
460.38528
461.38681
483.40249
Observed mass [m/z]

M6

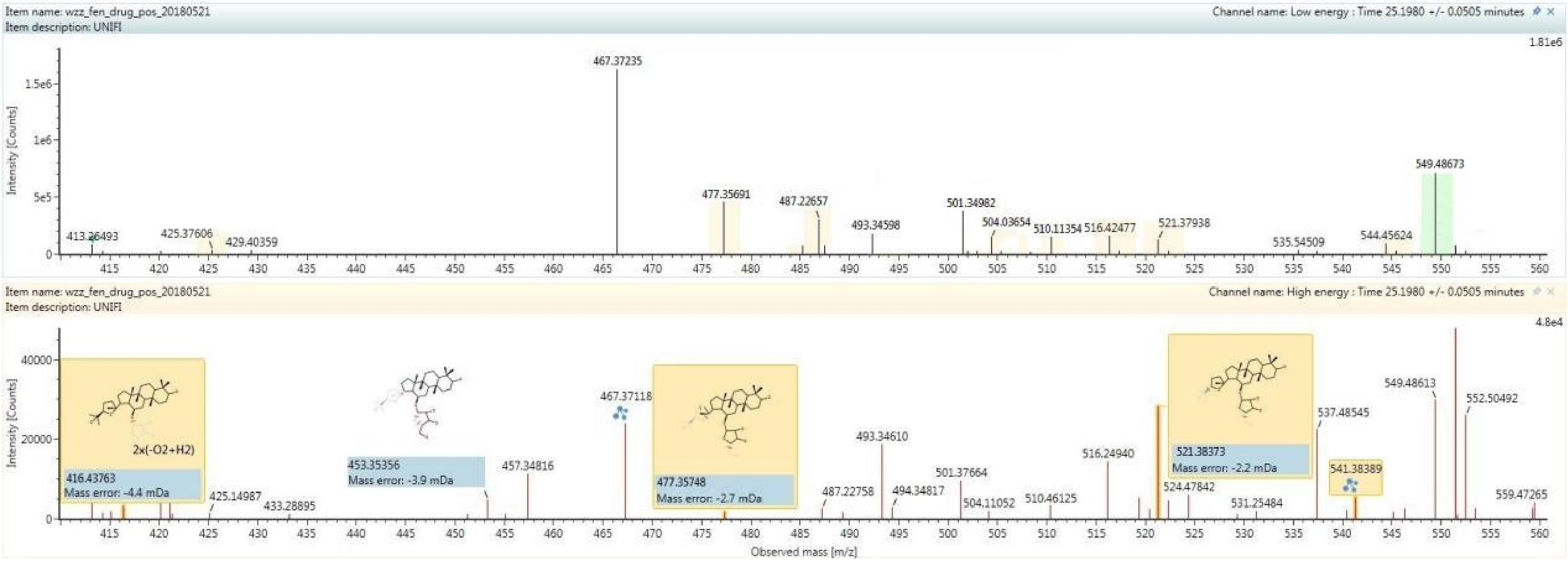

Item name: wzz_fen_drug_pos_20180521
Item description: UNIFI
Channel name: Low energy : Time 25.1980 +/- 0.0505 minutes
1.81e6
Intensity [Counts]
467.37235
549.48673
477.35691
487.22657
501.34982
493.34598
504.03654
510.11354
516.42477
521.37938
413.26493
425.37606
429.40359
535.54509
544.45624
Item name: wzz_fen_drug_pos_20180521
Item description: UNIFI
Channel name: High energy : Time 25.1980 +/- 0.0505 minutes
4.8e4
Intensity [Counts]
2x(-O2+H2)
416.43763
Mass error: -4.4 mDa
453.35356
Mass error: -3.9 mDa
467.37118
477.35748
Mass error: -2.7 mDa
521.38373
Mass error: -2.2 mDa
541.38389
425.14987
433.28895
457.34816
487.22758
493.34610
494.34817
501.37664
504.11052
510.46125
516.24940
524.47842
531.25484
537.48545
549.48613
552.50492
559.47265
Observed mass [m/z]

M7

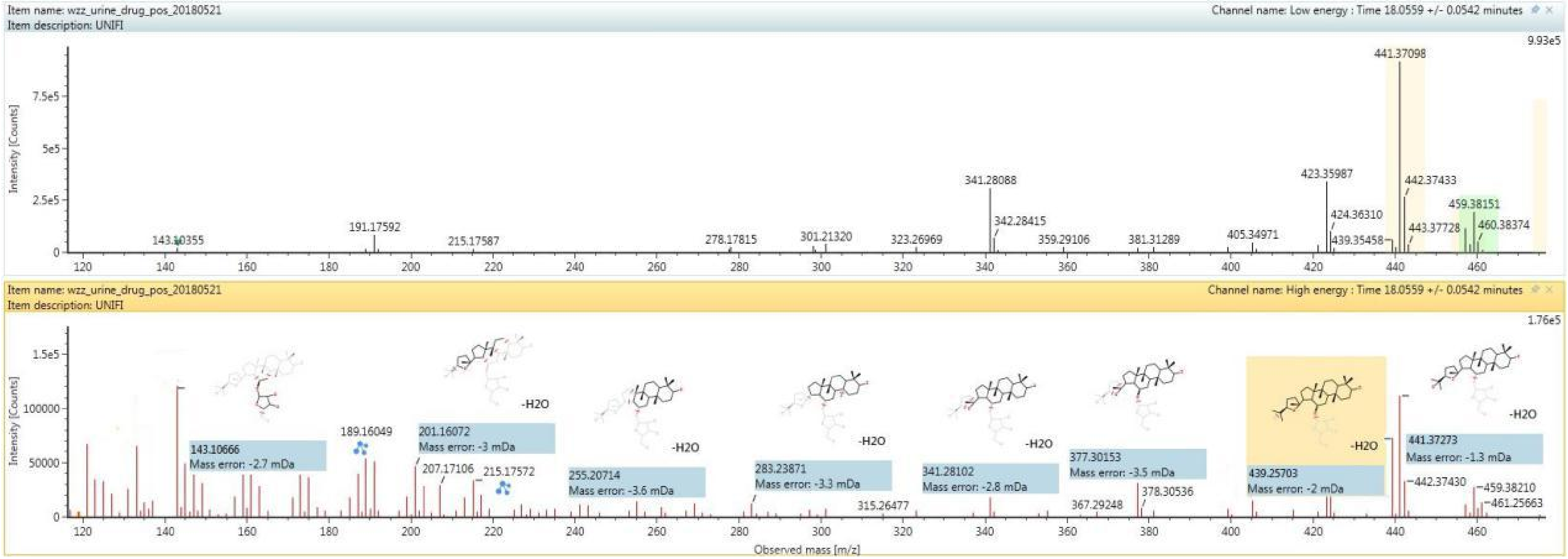

Item name: wzz_urine_drug_pos_20180521
Item description: UNIFI
Channel name: Low energy : Time 18.0559 +/- 0.0542 minutes
9.93e5
Intensity [Counts]
441.37098
423.35987
442.37433
341.28088
459.38151
342.28415
424.36310
460.38374
443.37728
191.17592
439.35458
405.34971
143.10355
215.17587
278.17815
301.21320
323.26969
359.29106
381.31289
Item name: wzz_urine_drug_pos_20180521
Item description: UNIFI
Channel name: High energy : Time 18.0559 +/- 0.0542 minutes
1.76e5
-H2O
143.10666
Mass error: -2.7 mDa
189.16049
201.16072
Mass error: -3 mDa
207.17106
215.17572
255.20714
Mass error: -3.6 mDa
283.23871
Mass error: -3.3 mDa
315.26477
341.28102
Mass error: -2.8 mDa
377.30153
Mass error: -3.5 mDa
367.29248
378.30536
439.25703
Mass error: -2 mDa
441.37273
Mass error: -1.3 mDa
442.37430
459.38210
461.25663
Observed mass [m/z]

M8

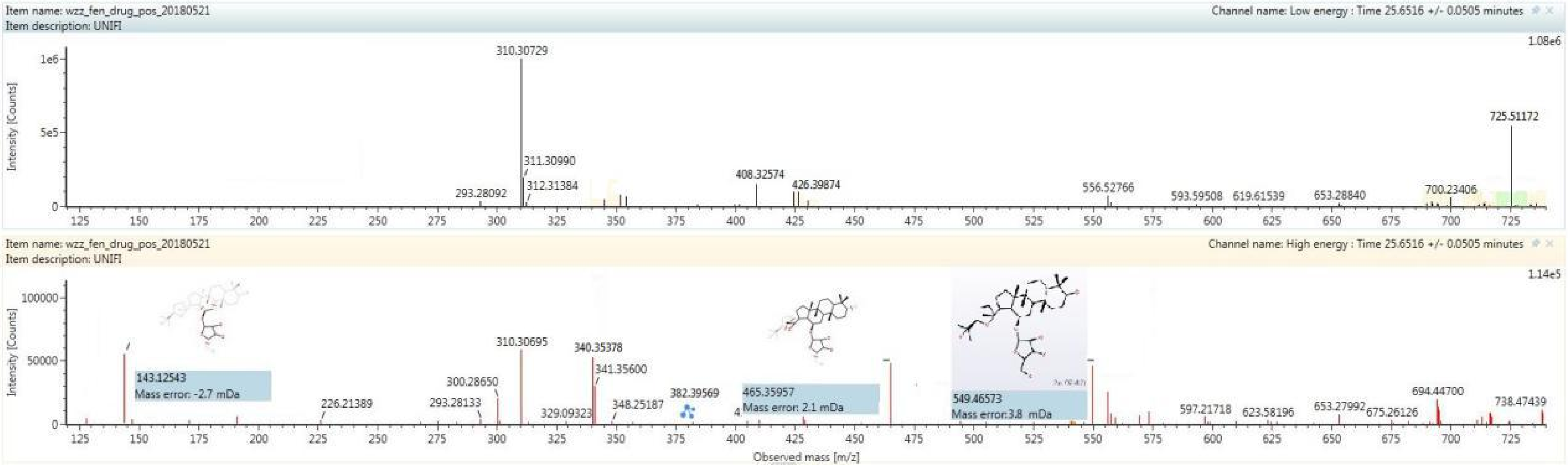

Item name: wzz_fen_drug_pos_20180521
Item description: UNIFI
Channel name: Low energy : Time 25.6516 +/- 0.0505 minutes
1.08e6
Intensity [Counts]
310.30729
725.51172
311.30990
312.31384
293.28092
408.32574
426.39874
556.52766
593.59508
619.61539
653.28840
700.23406
Item name: wzz_fen_drug_pos_20180521
Item description: UNIFI
Channel name: High energy : Time 25.6516 +/- 0.0505 minutes
1.14e5
143.12543
Mass error: -2.7 mDa
226.21389
300.28650
293.28133
310.30695
329.09323
340.35378
341.35600
348.25187
382.39569
465.35957
Mass error: 2.1 mDa
549.46573
Mass error:3.8 mDa
597.21718
623.58196
653.27992
675.26126
694.44700
738.47439
Observed mass [m/z]

M9

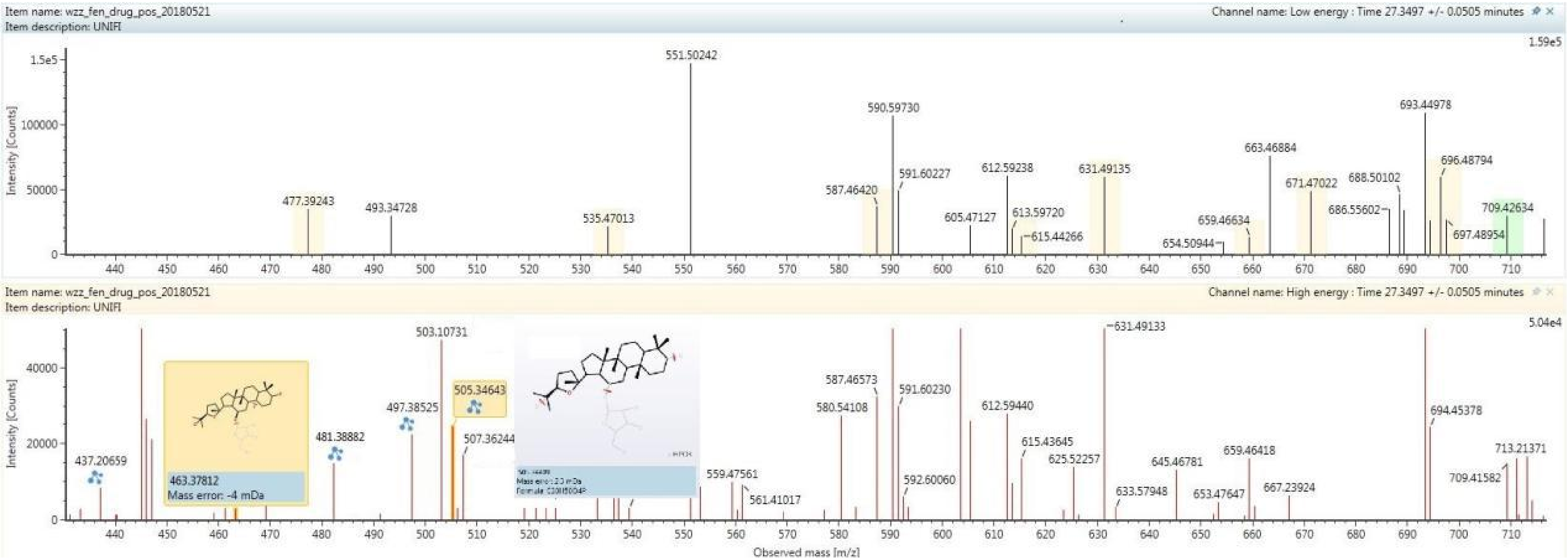

Item name: wzz_fen_drug_pos_20180521
Item description: UNIFI
Channel name: Low energy : Time 27.3497 +/- 0.0505 minutes
1.59e5
Intensity [Counts]
551.50242
590.59730
693.44978
663.46884
696.48794
612.59238
631.49135
587.46420
591.60227
671.47022
688.50102
477.39243
493.34728
535.47013
605.47127
613.59720
615.44266
659.46634
654.50944
686.55602
709.42634
697.48954
Item name: wzz_fen_drug_pos_20180521
Item description: UNIFI
Channel name: High energy : Time 27.3497 +/- 0.0505 minutes
5.04e4
503.10731
631.49133
587.46573
591.60230
505.34643
497.38525
481.38882
507.36244
437.20659
463.37812
Mass error: -4 mDa
580.54108
612.59440
694.45378
615.43645
625.52257
659.46418
645.46781
713.21371
709.41582
559.47561
592.60060
561.41017
633.57948
653.47647
667.23924
Observed mass [m/z]

M10

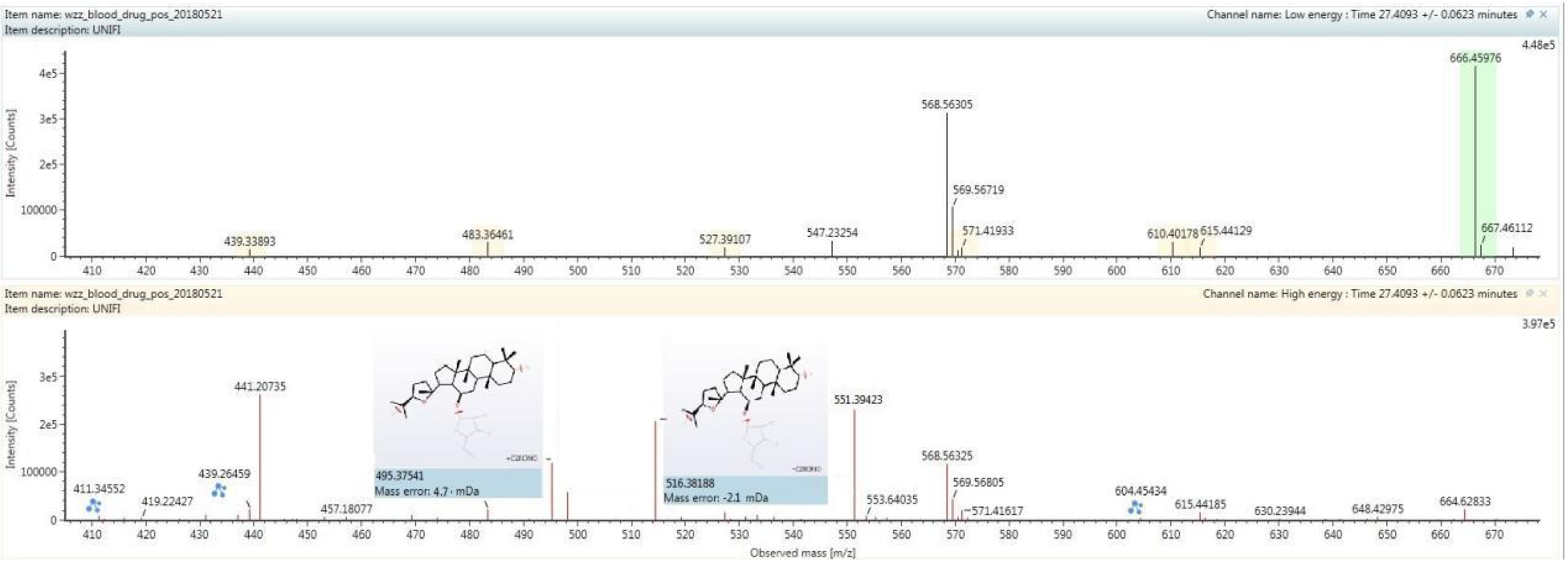

Item name: wzz_blood_drug_pos_20180521
Item description: UNIFI
Channel name: Low energy : Time 27.4093 +/- 0.0623 minutes
4.48e5
Intensity [Counts]
666.45976
568.56305
569.56719
571.41933
439.33893
483.36461
527.39107
547.23254
610.40178
615.44129
667.46112
Item name: wzz_blood_drug_pos_20180521
Item description: UNIFI
Channel name: High energy : Time 27.4093 +/- 0.0623 minutes
3.97e5
441.20735
495.37541
Mass error: 4.7 mDa
516.38188
Mass error: -2.1 mDa
551.39423
568.56325
569.56805
571.41617
553.64035
411.34552
419.22427
439.26459
457.18077
604.45434
615.44185
630.23944
648.42975
664.62833
Observed mass [m/z]

M11

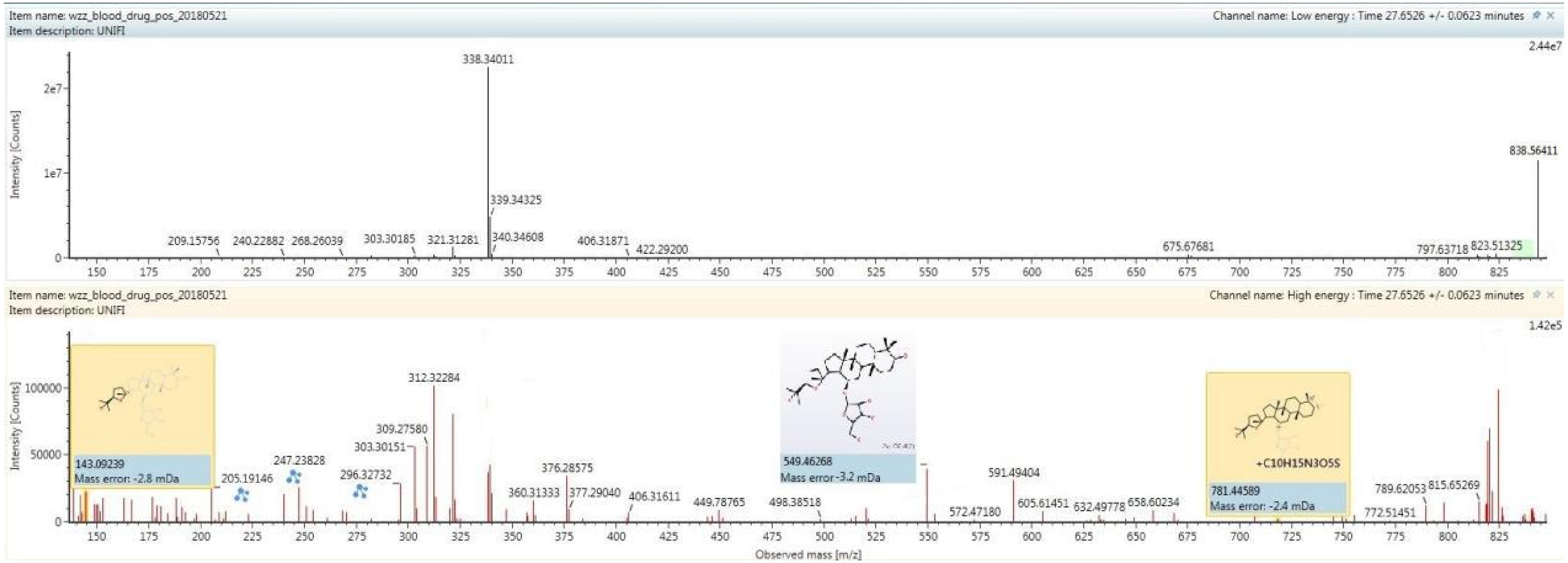

Item name: wzz_blood_drug_pos_20180521
Item description: UNIFI
Channel name: Low energy : Time 27.6526 +/- 0.0623 minutes
2.44e7
Intensity [Counts]
338.34011
838.56411
339.34325
340.34608
209.15756
240.22882
268.26039
303.30185
321.31281
406.31871
422.29200
675.67681
797.63718
823.51325
Item name: wzz_blood_drug_pos_20180521
Item description: UNIFI
Channel name: High energy : Time 27.6526 +/- 0.0623 minutes
1.42e5
312.32284
309.27580
303.30151
247.23828
296.32732
205.19146
376.28575
360.31333
377.29040
406.31611
449.78765
498.38518
143.09239
Mass error: -2.8 mDa
549.46268
Mass error:-3.2 mDa
591.49404
572.47180
605.61451
632.49778
658.60234
+C10H15N3O5S
781.44589
Mass error: -2.4 mDa
789.62053
815.65269
772.51451
Observed mass [m/z]

M12

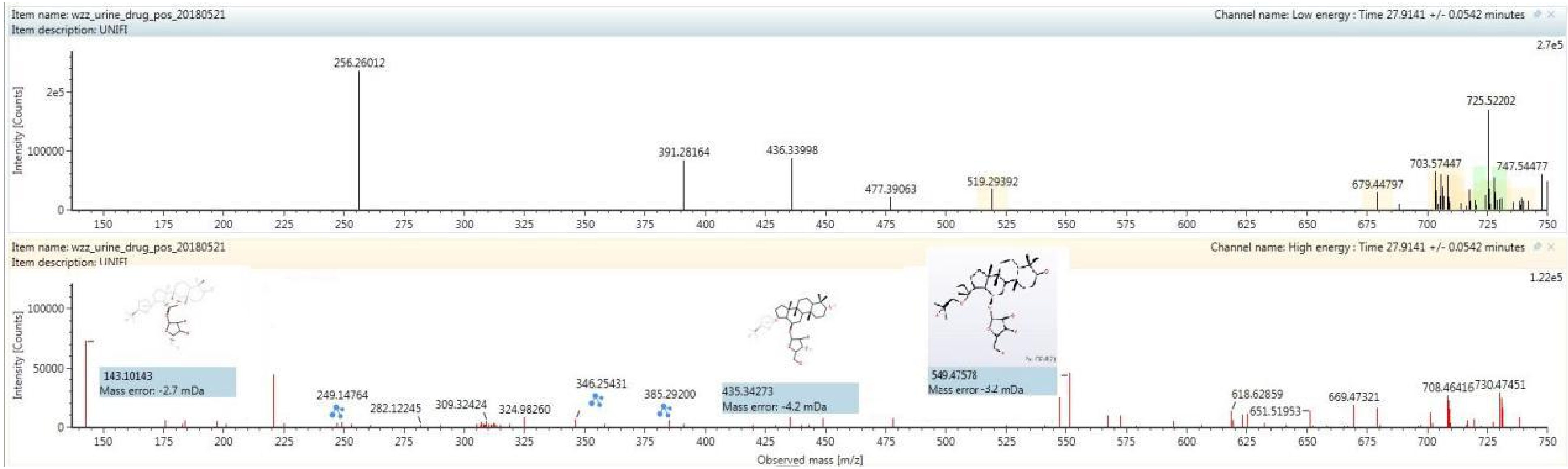

Item name: wzz_urine_drug_pos_20180521
Item description: UNIFI
Channel name: Low energy : Time 27.9141 +/- 0.0542 minutes
2.7e5
Intensity [Counts]
256.26012
391.28164
436.33998
477.39063
519.29392
679.44797
703.57447
725.52202
747.54477
Item name: wzz_urine_drug_pos_20180521
Channel name: High energy : Time 27.9141 +/- 0.0542 minutes
1.22e5
143.10143
Mass error: -2.7 mDa
249.14764
282.12245
309.32424
324.98260
346.25431
385.29200
435.34273
Mass error: -4.2 mDa
549.47578
Mass error -3.2 mDa
618.62859
651.51953
669.47321
708.46416
730.47451
Observed mass [m/z]

M13

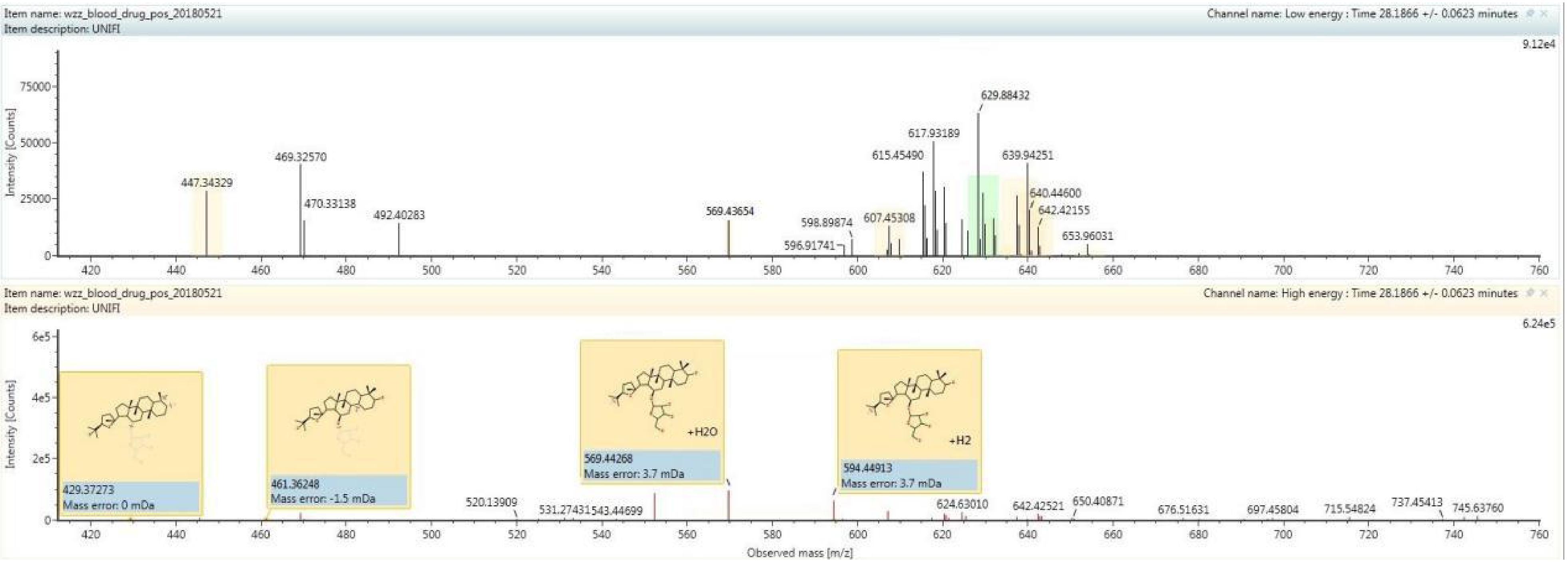

Item name: wzz_blood_drug_pos_20180521
Item description: UNIFI
Channel name: Low energy : Time 28.1866 +/- 0.0623 minutes
9.12e4
Intensity [Counts]
75000
50000
25000
0
447.34329
469.32570
470.33138
492.40283
569.43654
596.91741
598.89874
607.45308
615.45490
617.93189
629.88432
639.94251
640.44600
642.42155
653.96031
420 440 460 480 500 520 540 560 580 600 620 640 660 680 700 720 740 760
Item name: wzz_blood_drug_pos_20180521
Item description: UNIFI
Channel name: High energy : Time 28.1866 +/- 0.0623 minutes
6.24e5
Intensity [Counts]
6e5
4e5
2e5
0
429.37273
Mass error: 0 mDa
461.36248
Mass error: -1.5 mDa
+H2O
569.44268
Mass error: 3.7 mDa
+H2
594.44913
Mass error: 3.7 mDa
520.13909
531.27431
543.44699
624.63010
642.42521
650.40871
676.51631
697.45804
715.54824
737.45413
745.63760
420 440 460 480 500 520 540 560 580 600 620 640 660 680 700 720 740 760
Observed mass [m/z]
